# Ca_V_2.1 mediates presynaptic dysfunction induced by amyloid β oligomers

**DOI:** 10.1016/j.celrep.2025.115451

**Published:** 2025-03-23

**Authors:** Alexander F. Jeans, Zahid Padamsey, Helen Collins, William Foster, Sally Allison, Steven Dierksmeier, William L. Klein, Arn M.J.M. van den Maagdenberg, Nigel J. Emptage

**Affiliations:** 1 Department of Pharmacology, University of Oxford, Mansfield Road, Oxford OX1 3QT, UK; 2 Department of Neurobiology and Physiology, Northwestern University, Evanston, IL 60208, USA; 3 Departments of Human Genetics and Neurology, Leiden University Medical Centre, 2300 RC Leiden, the Netherlands

**Keywords:** Alzheimer's disease, neurodegeneration, Aβ oligomer, neurotransmitter release, synaptic vesicle, Ca2^+^ channel

## Abstract

Synaptic dysfunction is an early pathological phenotype of Alzheimer’s disease (AD) that is initiated by oligomers of amyloid β peptide (Aβ_o_s). Treatments aimed at correcting synaptic dysfunction could be beneficial in preventing disease progression, but mechanisms underlying Aβ_o_-induced synaptic defects remain incompletely understood. Here, we uncover an epithelial sodium channel (ENaC) - Ca_V_2.3 - protein kinase C (PKC) - glycogen synthase kinase-3β (GSK-3β) signal transduction pathway that is engaged by Aβ_o_s to enhance presynaptic Ca_V_2.1 voltage-gated Ca^2+^ channel activity, resulting in pathological potentiation of action-potential-evoked synaptic vesicle exocytosis. We present evidence that the pathway is active in human APP transgenic mice *in vivo* and in human AD brains, and we show that either pharmacological Ca_V_2.1 inhibition or genetic Ca_V_2.1 haploinsufficiency is sufficient to restore normal neurotransmitter release. These findings reveal a previously unrecognized mechanism driving synaptic dysfunction in AD and identify multiple potentially tractable therapeutic targets.

## Highlights

• Aβ oligomers drive Ca_V_2.1-mediated potentiation of presynaptic exocytosis
• Ca_V_2.1 function is upregulated via an ENaC-Ca_V_2.3-PKC-GSK-3β signaling pathway
• Ca_V_2.1-mediated potentiation of presynaptic exocytosis is found in AD model mice *in vivo*
• Presynaptic dysfunction can be rescued *in vivo* by lowering Ca_V_2.1 expression

## Graphical abstract

**Figure:**
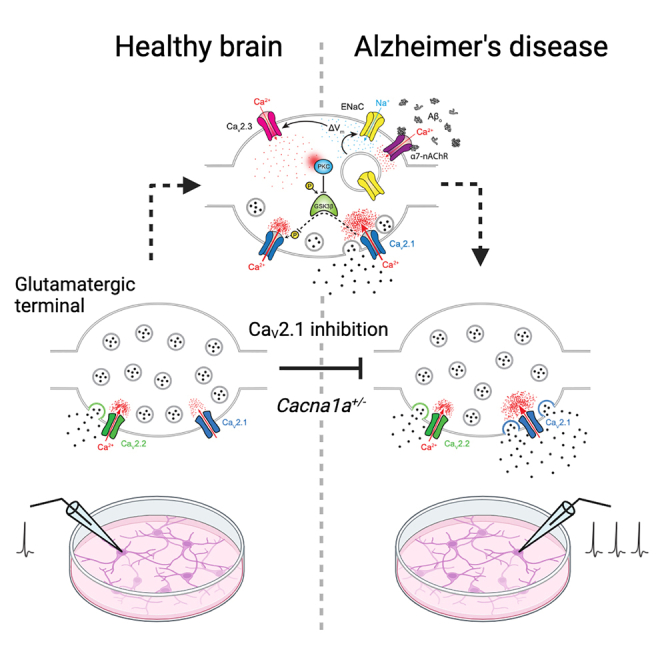

## Teaser

There is an urgent need for new targets for Alzheimer’s disease therapy. Jeans et al. identify a presynaptic signaling pathway engaged by Aβ oligomers that drives glutamatergic synaptic dysfunction via pathological enhancement of Ca_V_2.1 Ca^2+^ channel activity and synaptic vesicle exocytosis. Lowering Ca_V_2.1 expression restores normal synaptic function.

## Introduction

Alzheimer’s disease (AD) is increasing in prevalence in the aging population, and the identification of effective disease-modifying treatments remains a priority. Numerous lines of evidence suggest that amyloid β (Aβ) peptides, in particular Aβ oligomers (Aβ_o_s), are a key trigger of the synaptic toxicity and cognitive decline that characterize AD.^1^ Exposure to Aβ_o_s produces multiple effects on different cell types,^2^ with alterations in synaptic transmission being among the earliest observed. Aberrantly enhanced synaptic activity in excitatory neurons of the cortex and hippocampus is a particularly consistent early finding in both AD model systems and patients,^3^ although the underlying mechanisms remain a matter of debate.^4^^,^^5^

Since dysregulation of synaptic transmission is considered to be one of the key substrates of early cognitive decline in AD,^3^ elucidating the underlying mechanisms is of critical importance in order to devise effective new therapies. Accordingly, we set out to understand Aβ_o_-induced alterations in synaptic transmission in a variety of well-characterized AD model systems.

## Results

### Aβ_o_s induce a robust enhancement of evoked synaptic vesicle exocytosis in hippocampal neurons due to increased Ca^2+^ entry through presynaptic Ca_V_2.1 channels

We first studied the effects of acute applications of Aβ_o_s on synaptic transmission using patch-clamp recordings in cultured rat hippocampal pyramidal neurons. Although oligomers of a variety of sizes have been implicated in Aβ-mediated synaptotoxicity,^6^ we chose to focus on the effects of a well-characterized, pathologically relevant synthetic preparation of small oligomers of Aβ_1–42_ (Figure S1A), which have consistently been identified as the most synaptotoxic Aβ species and are present in AD-affected brains.^7^^,^^8^ Although there is a risk that synthetic oligomers might not replicate the posttranslational modifications of those isolated from native mouse or human brain, multiple studies have demonstrated that, in practice, their properties at both the structural and the pathological level align extremely well with those of their brain-derived counterparts.^6^

**Figure 1:**
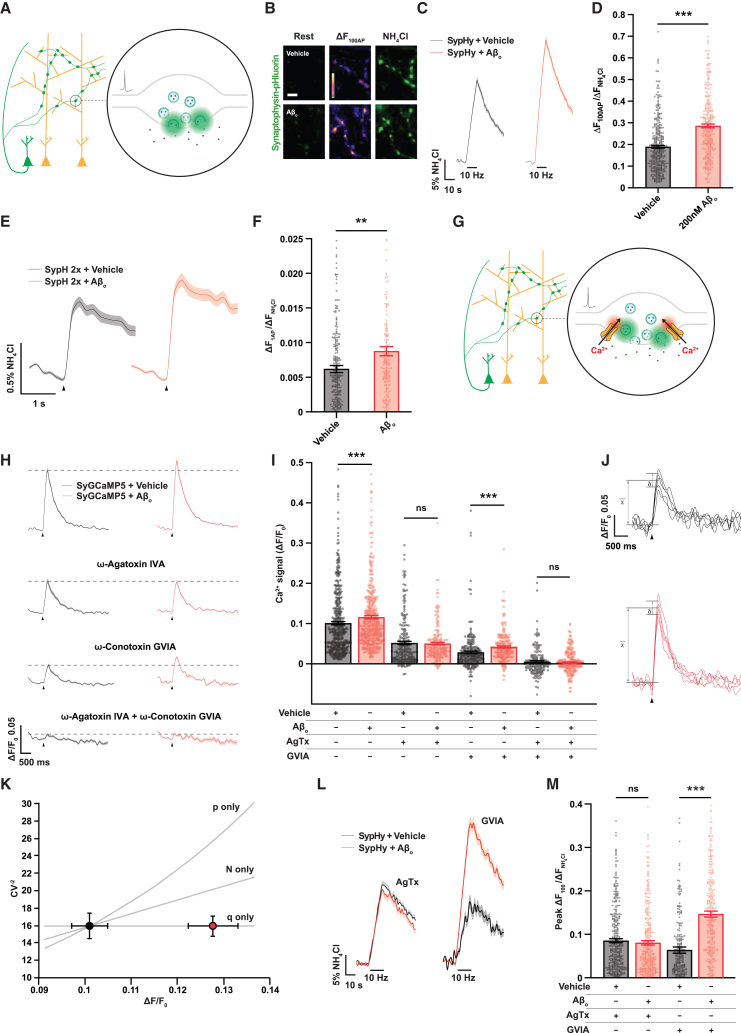
Aβ oligomers induce a robust enhancement of evoked synaptic vesicle exocytosis in hippocampal neurons due to enhanced Ca^2+^ entry through presynaptic Ca_V_2.1 channels (A) Imaging neurotransmitter release with a pHluorin probe. Synaptophysin-pHluorin (SypHy) is expressed on the luminal face of synaptic vesicles, where its fluorescence is quenched at intravesicular pH ∼5.5. Exocytosis exposes the luminal face of the vesicle to extracellular pH ∼7.4, unquenching SypHy fluorescence (green). (B) Representative images showing hippocampal neuronal boutons expressing SypHy and incubated for 2 h with either 200 nM Aβ_o_ or vehicle control. Middle column shows the increase in fluorescence after stimulation at 10 Hz for 10 s. Right-hand side shows maximal signal following unquenching of SypHy with NH_4_Cl, used for normalization as a control for SypHy expression level. Scale bar, 5 μm. (C) Average SypHy fluorescence traces during 10 Hz/10 s stimulation. (D) Mean peak amplitudes of 10 Hz/10 s responses (control, *n* = 364 synapses from seven coverslips, and 200 nM Aβ_o_, *n* = 323 synapses from seven coverslips). (E) Fluorescence traces showing the mean 10 trial average response of Aβ_o_- or vehicle control-incubated boutons expressing the enhanced sensitivity pHluorin SypH 2× to a single stimulus. Arrow indicates delivery of stimulus. (F) Mean amplitudes of (10 trial average) responses to a single stimulus (vehicle-treated control, *n* = 288 boutons from eight coverslips, and Aβ_o_, *n* = 199 boutons from seven coverslips). (G) Imaging presynaptic Ca^2+^ entry. The presynaptically targeted Ca^2+^ reporter SyGCaMP5 is a synaptophysin-GCaMP5 fusion protein that is expressed on the outer surface of synaptic vesicles where Ca^2+^ entry (red) via voltage-gated channels causes it to fluoresce (green). (H) Fluorescence traces showing the mean five trial average response of Aβ_o_- or vehicle control-incubated boutons expressing SyGCaMP5 to a single stimulus, with VGCC-blocking peptides ω-agatoxin IVA (Ca_V_2.1) and/or ω-conotoxin GVIA (Ca_V_2.2) present as indicated. Arrow indicates delivery of stimulus. (I) Mean peak amplitudes of (five trial average) Ca^2+^ signals obtained under the conditions indicated (vehicle, *n* = 477 boutons from twelve coverslips; Aβ_o_, *n* = 505 boutons from fourteen coverslips; vehicle + ω-agatoxin IVA, *n* = 225 boutons from five coverslips; Aβ_o_ + ω-agatoxin IVA, *n* = 207 boutons from six coverslips; vehicle + ω-conotoxin GVIA, *n* = 258 boutons from six coverslips; Aβ_o_ + ω-conotoxin GVIA, *n* = 240 boutons from five coverslips; vehicle + ω-agatoxin IVA + ω-conotoxin GVIA, *n* = 170 boutons from eight coverslips; and Aβ_o_ + ω-agatoxin IVA + ω-conotoxin GVIA, *n* = 268 boutons from ten coverslips). ANOVA with *post hoc* t test and Sidak correction. (J) Optical fluctuation analysis of Ca^2+^ responses using data from vehicle- and Aβ_o_-treated boutons represented in (I). Analysis of trial-to-trial fluctuations in responses enables differences in mean response between groups to be attributed to changes in the number of VGCCs (N), their open probability (p), or their unitary Ca^2+^ currents (q). Representative five trial sets of traces are shown ($\bar{x}$, mean; δ, standard deviation of Δ[Ca^2+^]; arrowheads indicate delivery of stimulus). (K) Relationship between the mean of the inverse squared coefficient of variation (CV^−2^) and the mean amplitude of responses in each condition. The gray lines are predictions of what would be observed if the change in Ca^2+^ response amplitude were explained solely by changes in N, p, or q. The Bayesian information criterion (BIC) confirmed that, of the three possible models, variation in q provided the best fit to the experimental data (ΔBIC for both p vs. q and N vs. q > 200 in favor of q). (L) Ca_V_2.1 (ω-agatoxin IVA sensitive) but not Ca_V_2.2 (ω-conotoxin GVIA sensitive) VGCCs are necessary for Aβ_o_-enhanced exocytosis. Average SypHy fluorescence traces obtained in response to 100 stimuli delivered at 10 Hz in the presence of either ω-agatoxin IVA (left) or ω-conotoxin GVIA (right) are shown. (M) Mean peak amplitudes of responses to 100 stimuli delivered at 10 Hz in ω-agatoxin IVA or ω-conotoxin GVIA (control + agatoxin, *n* = 382 boutons from seven coverslips; Aβ_o_ + agatoxin, *n* = 372 boutons from seven coverslips; control + conotoxin, *n* = 259 boutons from seven coverslips; and Aβ_o_ + conotoxin, *n* = 322 boutons from seven coverslips). Shading or error bars represent ± SEM. ^∗∗^*p* < 0.01, ^∗∗∗^*p* < 0.0001, and ns, non-significant.

A 2 h incubation with 200 nM Aβ_o_ induced an increase in overall excitatory glutamatergic synaptic activity, while inhibitory activity was unchanged (Figures S1B–S1D). This could potentially be explained by changes in either the duration or the amplitude of action potentials in excitatory glutamatergic neurons or a change in neuronal excitability. However, we found that none of these parameters were altered by Aβ_o_ treatment (Figures S1E–S1I), although we note that these recordings were made at the soma, and it remains formally possible that the voltage waveform at the presynaptic terminal, which is not feasible to record directly, could be different. We then focused on the regulation of neurotransmitter release at the presynaptic terminal, which we imaged in neurons expressing a high-resolution fluorescent reporter of synaptic vesicle exocytosis, synaptophysin-pHluorin (SypHy)^9^ (Figure 1A). Incubation with Aβ_o_s produced a >40% potentiation of exocytosis in response to a 10 Hz stimulus train (Figures 1B–1D), an effect that was similar across a wide range of Aβ_o_ concentrations (Figures S2A and S2B). To confirm that this effect does not reflect changes at inhibitory terminals, we expressed a pHluorin fused to the vesicular GABA transporter (vGAT-pHluorin) under the control of the GAD67 promoter, which restricts expression to GABAergic synapses.^10^ This showed no change in exocytosis following Aβ_o_ incubation (Figures S2C–S2E).

During the repetitive action potential firing elicited by stimulus trains, the total amount of exocytosis at each synapse is regulated by multiple parameters, including the rate of replenishment of the pool of available synaptic vesicles and the probability that any single action potential will drive the exocytosis of one or more release-competent vesicles, known as the probability of release. Therefore, to refine possible targets of Aβ_o_ action, we examined exocytosis in response to single action potential stimuli directly using SypHluorin 2× (SypH 2×), a derivative of SypHy with enhanced sensitivity.^11^ The average single-stimulus SypH 2× response measured over 10 trials was around 40% greater in neurons exposed to Aβ_o_s, indicating that this treatment robustly enhances probability of release (Figures 1E and 1F).

The probability of neurotransmitter release at hippocampal boutons is set by action-potential-evoked Ca^2+^ influx through presynaptic voltage-gated Ca^2+^ channels (VGCCs).^12^ We studied boutonal Ca^2+^ transients using the presynaptically localized Ca^2+^ reporter SyGCaMP5 (Figure 1G). Focusing on single-stimulus-evoked events, since these lie within the linear range of this probe,^13^ we observed an approximately 10% increase in Ca^2+^ influx in the presence of Aβ_o_s (Figures 1H and 1I). We used optical fluctuation analysis, which analyzes trial-to-trial variation in boutonal Ca^2+^ transients to determine whether changes in Ca^2+^ influx are due to changes in the number or properties of VGCCs.^14^ This demonstrated that the enhanced presynaptic Ca^2+^ influx was due to an increase in unitary Ca^2+^ channel currents rather than the number of available channels or their opening probability (Figures 1J and 1K). In addition to altered Ca^2+^ influx, changes in probability of release can also be driven by changes in the proximity of VGCCs to the neurotransmitter release machinery.^12^ We tested this using the slow Ca^2+^ chelator EGTA. If the chelator is present in excess, and therefore non-saturable, the percentage inhibition of exocytosis will depend on the distance between the Ca^2+^ channel pore and the release sensor (synaptotagmin), as well as the chelator’s Ca^2+^ binding kinetics.^15^ Importantly, it will not be sensitive to the total Ca^2+^ influx through the channel,^15^ which changes following Aβ_o_ exposure. We found that a brief incubation in a set concentration of cell-permeative EGTA-AM (200 μm for 90 s) depressed exocytosis in response to a single stimulus to a lesser extent following Aβ_o_ incubation (Figures S2F and S2G), indicating tighter physical coupling of VGCCs to the release machinery. Together, these changes can account fully for the enhancement of synaptic vesicle exocytosis by Aβ_o_s, particularly given the highly non-linear, co-operative relationship between Ca^2+^ influx and vesicular neurotransmitter release at mammalian hippocampal synapses.^16^

Ca^2+^ influx at glutamatergic hippocampal terminals is predominantly dependent upon a combination of Ca_V_2.2 and Ca_V_2.1 VGCCs,^17^ and we used the specific Ca^2+^-channel-blocking peptides ω-conotoxin GVIA (Ca_V_2.2) and ω-agatoxin IVA (Ca_V_2.1) to dissect the relative contribution of these channels to the Aβ_o_-mediated enhancement of Ca^2+^ entry. We found that the enhancement is abolished only in the presence of ω-agatoxin IVA (Figures 1H and 1I), indicating that it is mediated by Ca_V_2.1 but not Ca_V_2.2 VGCCs. There was no difference in the Ca^2+^ signal following Aβ_o_ incubation when both Ca_V_2.1 and Ca_V_2.2 were blocked, indicating that non-Ca_V_2.1/Ca_V_2.2 Ca^2+^ sources do not contribute significantly (Figures 1H and 1I). To confirm that enhanced Ca^2+^ entry specifically mediated via Ca_V_2.1 drives the increase in synaptic vesicle exocytosis, we used VGCC-blocking peptides with SypHy-expressing neurons to show that Ca_V_2.1, but not Ca_V_2.2, channels are necessary for Aβ_o_-mediated potentiation of the response to a 10 Hz stimulus train (Figures 1L and 1M).

### Exocytosis is potentiated via a presynaptic ENaC-Ca_V_2.3-protein kinase C (PKC) signaling axis

**Figure 2:**
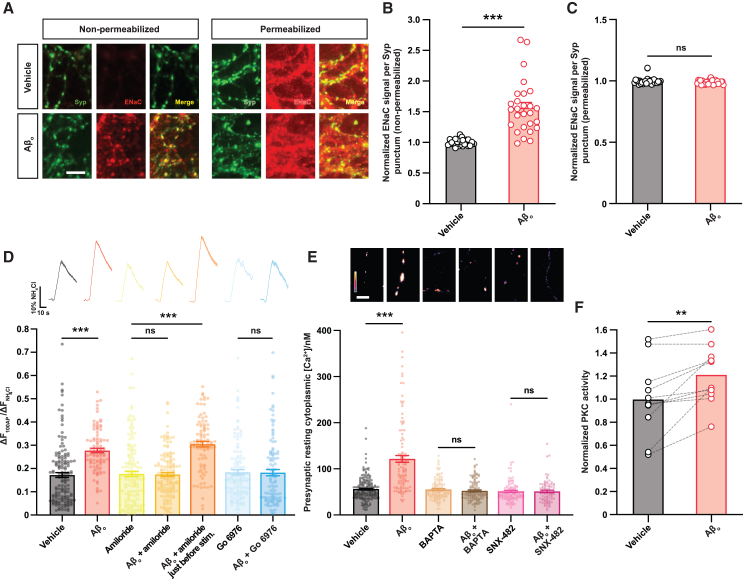
Exocytosis is potentiated via a presynaptic ENaC-Ca_V_2.3-PKC signaling axis (A) Representative images of ENaC immunofluorescence following treatment as indicated. Labeling of non-permeabilized cells reveals only membrane-inserted surface ENaCs, while permeabilized cells were treated with detergent to ensure that the antibody was able to penetrate the entire cell. To examine presynaptic ENaCs specifically, only labeling overlapping with the presynaptic marker synaptophysin (Syp) was assessed. Scale bar, 5 μm. (B) Normalized ENaC labeling intensity per synaptophysin-positive punctum in non-permeabilized cells (*n* ≥ 27 cells per condition). (C) Normalized ENaC labeling intensity per synaptophysin-positive punctum in permeabilized cells (*n* ≥ 28 cells per condition). Note that these signals were not saturated. (D) Amiloride, a blocker of the Na^+^ leak channel ENaC, and Go 6976, a protein kinase C antagonist, rescue the effect of Aβ_o_s on neurotransmitter release in response to a 100 AP/10 Hz stimulus in SypHy-expressing neuronal cultures. The rescue effect of amiloride is not observed if the drug is applied immediately before stimulation, suggesting that ENaCs regulate Ca_V_2.1 via a signaling cascade rather than directly modulating Ca_V_2.1 gating via constitutive depolarization, which would require the ENaC to remain open during stimulation. Responses are normalized to maximal NH_4_Cl signal, and mean peak amplitudes are shown. Average fluorescence traces are above the bars (vehicle-treated control, *n* = 150 boutons from five coverslips; Aβ_o_, *n* = 95 boutons from seven coverslips; amiloride, *n* = 150 boutons from six coverslips; Aβ_o_ + amiloride, *n* = 146 boutons from five coverslips; Aβ_o_ + amiloride immediately before stimulation, *n* = 90 boutons from five coverslips; Go 6976, *n* = 118 boutons from five coverslips; and Aβ_o_ + Go 6976, *n* = 113 boutons from five coverslips). ANOVA with *post hoc* t test and Sidak correction. (E) A resting boutonal cytoplasmic [Ca^2+^] rise following Aβ_o_ exposure is abolished by either the Ca_V_2.3 inhibitor SNX-482 or chelation of extracellular Ca^2+^ with BAPTA. The basal signal from hippocampal neuronal boutons expressing SyCCaMP5 was used along with a maximal signal induced by treatment with the Ca^2+^ ionophore ionomycin to determine resting cytoplasmic [Ca^2+^]. Mean values following treatment as indicated are shown (vehicle-treated control, *n* = 194 synapses from six coverslips; Aβ_o_, *n* = 112 synapses from six coverslips; BAPTA, *n* = 110 synapses from six coverslips; Aβ_o_ + BAPTA, *n* = 116 synapses from six coverslips; SNX-482, *n* = 117 synapses from six coverslips; and Aβ_o_ + SNX-482, *n* = 81 synapses from five coverslips). ANOVA with *post hoc* t test and Sidak correction. Image above each bar shows basal SyGCaMP5 signal from a representative field. Scale bar, 5 μm. (F) Synaptosomes were prepared from individual hippocampal neuronal cultures, then each synaptosome preparation was divided into two and subjected to treatments as indicated. PKC activity was then assessed in synaptosomal lysates using a specific ELISA-based assay (*n* = 11 cultures for all conditions). Paired t test. Shading or error bars represent ± SEM. ^∗∗^*p* < 0.01, ^∗∗^^∗^*p* < 0.0001, and ns, non-significant.

We set out to identify the molecular signaling pathway engaged by Aβ_o_s to enhance Ca_V_2.1 function and synaptic vesicle exocytosis. We previously reported that a similar specific upregulation of Ca_V_2.1 function without change to Ca_V_2.2 underlies the potentiation of evoked synaptic vesicle exocytosis in the context of homeostatic synaptic plasticity (HSP),^18^ a physiological process that regulates the strength of synapses to maintain activity in neuronal networks within set bounds.^19^ Potentiation of synaptic vesicle exocytosis by HSP at both *Drosophila* and mammalian synapses requires the insertion of a preexisting intracellular pool of the epithelial sodium channel (ENaC) into the presynaptic membrane,^20^^,^^21^ and we hypothesized that this might be a conserved mechanism for Ca_V_2.1-dependent regulation of presynaptic strength that could also underlie the presynaptic effects of Aβ_o_s. Using immunofluorescence on non-permeabilized and permeabilized neurons in culture to disclose surface and total presynaptic ENaCs, respectively, we found that, while the total presynaptic ENaC level did not change over the time course of the experiments, there was robust insertion of channels into the presynaptic membrane in response to Aβ_o_s (Figures 2A–2C). To then confirm that ENaC activity is necessary for the ability of Aβ_o_s to enhance exocytosis, we showed that the specific ENaC blocker amiloride^22^ was able to rescue the effects of Aβ_o_ treatment in SypHy-expressing neurons (Figure 2D). In these and subsequent experiments, we chose to assay Ca_V_2.1-driven changes in synaptic vesicle exocytosis rather than examining Ca^2+^ influx directly because the power law relationship between these two parameters^16^ lends experimental measurements of exocytosis much greater sensitivity and robustness.

ENaCs carry a Na^+^ leak current that causes a modest, chronic depolarization of the bouton.^21^ This could directly augment action-potential-evoked depolarization and thereby Ca^2+^ influx through VGCCs, or, alternatively, ENaCs could enhance Ca_V_2.1 function indirectly via recruitment of a signaling pathway. We sought to distinguish between these two possible modes of action by performing a second set of ENaC blockade experiments in which amiloride was not present during the Aβ_o_ incubation, allowing potential signaling events downstream of ENaC depolarization to take place, but was applied immediately before stimulation and imaging and stimulation to abolish any direct contribution of ENaC-mediated depolarization to exocytosis. This failed to rescue the effects of Aβ_o_ (Figure 2D), suggesting that signaling downstream of channel insertion is necessary. This result is in keeping with the specific enhancement of Ca_V_2.1, but not Ca_V_2.2, function by ENaC insertion; if direct effects of depolarization on channel function were responsible, a similar enhancement of both VGCC types would be expected, since they have similar gating properties.^23^

Chronic, subthreshold depolarization similar to that mediated by ENaCs can initiate intracellular signaling by mobilizing intra- or extracellular Ca^2+^ sources to raise cytoplasmic [Ca^2+^]. Resting [Ca^2+^] was determined at SyGCaMP5-expressing boutons from the GCaMP5 signal,^24^ and we found that Aβ_o_s elicited a clear [Ca^2+^] increase (Figure 2E). We considered possible Ca^2+^ sources that could support this rise, including intracellular stores and presynaptic VGCCs, particularly subtypes with a low activation voltage that are more efficiently opened by subthreshold depolarization, such as Ca_V_3 (T-type) or Ca_V_2.3 (R-type)^23^; we focused on the latter, as Ca_V_3 is not expressed at glutamatergic hippocampal terminals.^25^ We found that incubation with either BAPTA, to chelate extracellular Ca^2+^, or the Ca_V_2.3-blocking peptide SNX-482 abolished the Aβ_o_-mediated rise in resting cytoplasmic [Ca^2+^], indicating that intracellular stores are not sufficient for this, but Ca_V_2.3 is required (Figure 2E). We then directly linked Ca_V_2.3 to the effects of Aβ_o_s on synaptic vesicle exocytosis by using SypHy-expressing neurons to show that SNX-482 abolishes potentiation of the response to a 10 Hz stimulus train (Figure S2H). Previous work has suggested that Aβ_o_s can activate presynaptic mGluR5 receptors,^26^ which could potentially also contribute to a rise in cytoplasmic [Ca^2+^]. We therefore tested the potential involvement of this mechanism, finding that mGluR5 blockade with the specific antagonist SIB-1757 did not prevent the increase in exocytosis seen following Aβ_o_ exposure (Figure S2H).

Intracellular Ca^2+^ rises can activate Ca^2+^-sensing proteins, which in turn can trigger functional changes in a variety of substrates. Aside from the exocytotic Ca^2+^ sensors that mediate excitation-secretion coupling, the predominant Ca^2+^-sensing proteins expressed presynaptically are PKC^27^ and calmodulin (CaM), which directly regulates the activity of a wide variety of proteins, including CaM kinase II, calcineurin, and Ca_V_2.1.^28^ However, we have shown that Aβ_o_ exposure produces a rise in presynaptic cytoplasmic [Ca^2+^] of approximately 60 nM (Figure 2E), far short of the [Ca^2+^] elevation required to activate CaM, which is well into the micromolar range.^29^ PKC, on the other hand, is activated by nanomolar presynaptic [Ca^2+^] rises very comparable to what we have demonstrated.^30^ To ask whether Aβ_o_s activate presynaptic PKC, we prepared synaptosomes, which comprise the complete presynaptic terminal along with the postsynaptic density, from cultured hippocampal neurons and subjected these to various treatments before assessing PKC activity in synaptosomal lysates with a specific ELISA-based assay. We found that Aβ_o_ treatment increased PKC activity in the synaptosomes (Figure 2F), and this effect was blocked by the inclusion of SNX-482 (Figure S2I), corroborating an essential upstream role of Ca_V_2.3 in Aβ_o_-linked PKC activation. Finally, to confirm that PKC activity is necessary for the ability of Aβ_o_s to enhance exocytosis, we applied the PKC inhibitor Go 6976 to SypHy-expressing neurons, showing that it rescued the enhancement of synaptic vesicle exocytosis by Aβ_o_s, consistent with an essential role for PKC in this process (Figure 2D). Since resting presynaptic [Ca^2+^] varies even among boutons on the same axon,^31^ this mechanism could be consistent with independent regulation of release probability at individual synapses.

### GSK-3β is required for the enhancement of exocytosis by Aβ_o_s

**Figure 3:**
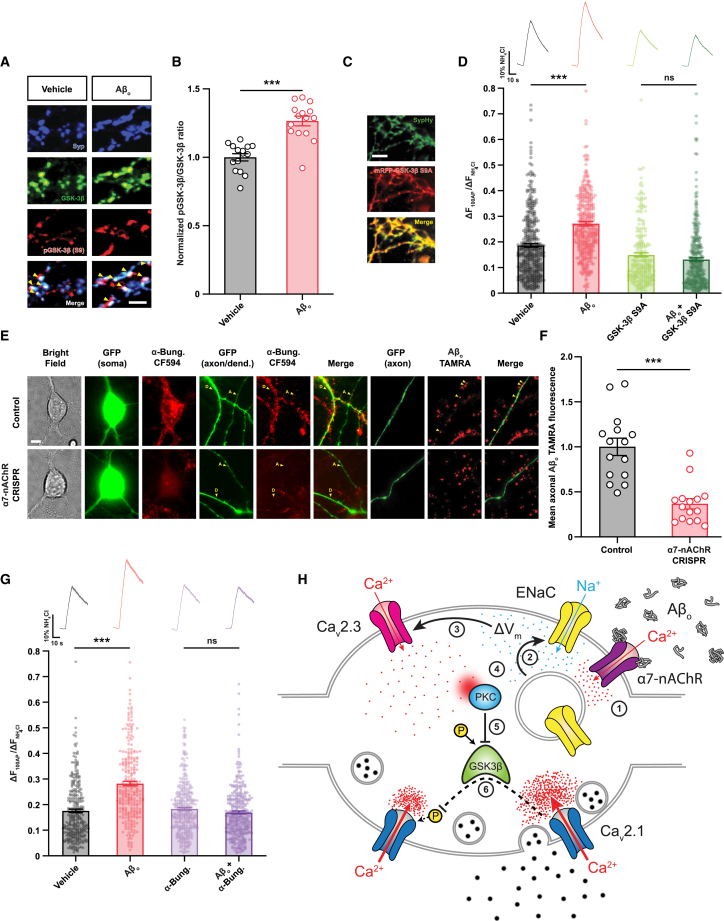
Potentiation of exocytosis by Aβ_o_s requires GSK-3β and α7-nAChR (A) Representative fields showing immunofluorescence for total GSK-3β and GSK-3β that has been inactivated by phosphorylation of the serine 9 residue. To examine presynaptic GSK-3β specifically, labeling overlapping with synaptophysin (Syp) was assessed. Bottom shows merged images in which areas of overlapping signal from all three channels appear white (arrowheads). Scale bar, 5 μm. (B) The fraction of GSK-3β that has undergone inactivating phosphorylation at S9 is represented as the S9 phosphorylated:total GSK-3β signal intensity ratio normalized to control average (*n* = 15 fields from five cultures per condition). (C) mRFP-GSK-3β (S9A), a constitutively active form of the enzyme insensitive to negative regulatory phosphorylation, was expressed in neurons along with SypHy to probe the requirement for negative regulation of GSK-3β in Aβ_o_-enhanced synaptic vesicle exocytosis. Representative fields illustrating co-localized signals from SypHy and RFP are shown (scale bar, 5 μm). (D) Mean peak amplitudes of responses to a 10 Hz/10 s stimulus normalized to maximal NH_4_Cl response. Expression of constitutively active GSK-3β (S9A) reduces the peak exocytotic response in vehicle-treated cultures and abolishes the effect of Aβ_o_s. Average fluorescence traces above bars (control, *n* = 412 boutons from six coverslips; Aβ_o_, *n* = 374 boutons from seven coverslips; mRFP-GSK-3β S9A, *n* = 230 boutons from seven coverslips; and mRFP-GSK-3β S9A + Aβ_o_, *n* = 349 boutons from seven coverslips). ANOVA with *post hoc* t test and Sidak correction. (E) Representative images showing hippocampal neurons expressing CRISPR-Cas9 knockout constructs targeting either the α7 subunit of nAChR or firefly luciferase as a negative control. Both constructs also include a GFP marker. Images left to right show different views of labeled neurons incubated with either the fluorescently tagged high-affinity α7-nAChR ligand α-bungarotoxin CF594, demonstrating the loss of surface protein in soma, dendrites (D), and axons (A) of cells expressing the α7-nAChR CRISPR construct, or Aβ_o_s tagged with the fluorophore tetramethyl rhodamine (TAMRA), showing punctate binding to morphologically identified axons (arrowheads) in control cells that is lost in α7-nAChR-knockout axons. Scale bar, 10 μm. (F) Normalized mean axonal fluorescence intensity in control and α7-nAChR-knockout neurons incubated with Aβ_o_ TAMRA (*n* = 15 neurons for both conditions). (G) The α7-nAChR antagonist α-bungarotoxin rescues Aβ_o_-induced enhancement of synaptic vesicle exocytosis. Mean peak amplitudes of SypHy responses to a 100 AP/10 Hz stimulus train following the indicated treatments, with average traces above bars (vehicle-treated control, *n* = 324 synapses from seven coverslips; Aβ_o_, *n* = 273 synapses from seven coverslips; vehicle + α-bungarotoxin, *n* = 392 boutons from seven coverslips; and Aβ_o_ + α-bungarotoxin, *n* = 346 boutons from seven coverslips). (H) Mechanistic model for enhancement of neurotransmitter release by Aβ_o_s. Arrows and blunted lines indicate activating and inhibitory processes, respectively, and a dashed line indicates inhibition by Aβ_o_s. Our data support a model in which pathological Aβ_o_s elicit presynaptic Ca^2+^ entry via α7-nAChR (1) to drive insertion of an intracellular pool of ENaCs into the presynaptic membrane (2). This causes an increase in Na^+^ influx and a resulting change in presynaptic resting membrane potential ΔV_m_, which enhances Ca_V_2.3 VGCC opening to elevate resting [Ca^2+^] at the presynaptic terminal (3). This [Ca^2+^] increase activates protein kinase C (PKC) (4), a negative regulator of GSK-3β, increasing the fraction of phosphorylated, inactive GSK-3β (5) and thereby inhibiting GSK-3β-mediated negative regulation of Ca_V_2.1 function (6). The result is increased Ca^2+^ influx via Ca_V_2.1 channels, with enhancement of evoked neurotransmitter release. Shading or error bars represent ± SEM. ^∗∗∗^*p* < 0.0001 and ns, non-significant.

Next, we sought a mechanistic link between PKC activation and enhanced Ca_V_2.1 function and exocytosis. Our data show that following exposure to Aβ_o_s, the increase in Ca_V_2.1-driven neurotransmitter release is due to changes in both unitary Ca^2+^ currents and physical coupling of the channels to release machinery. In VGCCs of the Ca_V_2 family, both can be achieved via the phosphorylation of a partially conserved intracellular amino acid sequence known as the synprint (synaptic protein interaction) site, which interacts directly with the soluble *N*-ethylmaleimide-sensitive factor attachment protein receptor (SNARE) proteins that mediate synaptic vesicle exocytosis.^12^ While a number of kinases are known to phosphorylate the Ca_V_2.2 synprint site,^12^ the Ca_V_2.1 synprint sequence is different and just one Ca_V_2.1 synprint kinase has been reported to date, glycogen synthase kinase-3β (GSK-3β), which phosphorylates Ca_V_2.1 to suppress both Ca^2+^ currents and SNARE protein binding.^32^ It is itself inactivated via phosphorylation at residue serine 9 by a variety of kinases, including PKC.^33^ To test its involvement, we first used immunofluorescence to assess the proportion of GSK-3β within hippocampal presynaptic terminals that is S9 phosphorylated, and therefore inactivated, and found that this increases following Aβ_o_ treatment (Figures 3A and 3B). We then co-expressed SypHy together with a constitutively active mutant form of GSK-3β that is not subject to phosphoregulation at serine 9^34^ and showed that Aβ_o_s were no longer able to enhance exocytosis (Figures 3C and 3D), indicating that negative phosphoregulation of GSK-3β is necessary for this effect. To place GSK-3β in our proposed pathway, we again immunostained Aβ_o_-treated hippocampal neurons for S9-phosphorylated and total GSK-3β, this time including specific inhibitors of ENaCs, Ca_V_2.3, or PKC. As before, we found that Aβ_o_ treatment increased the proportion of phospho-GSK-3β within presynaptic terminals, but that this effect was prevented in the presence of any of the inhibitors (Figure S3A), confirming that GSK-3β lies mechanistically downstream of each of them. Finally, we considered the question of whether an opposing phosphatase acts alongside GSK-3β in the regulation of Ca_V_2.1 by Aβ_o_s. Very little is known about Ca_V_2.1 phosphatases, but it has been shown that the synprint site of the related Ca_V_2.2 is dephosphorylated by multiple phosphatases, including the canonical cellular phosphatases PP1 and PP2a.^35^ We therefore undertook a preliminary investigation of their involvement in this pathway using a pharmacological inhibitor of PP1 and PP2a, tautomycin. We found that tautomycin alone resulted in a small but significant reduction in exocytosis in SypHy-expressing neurons, while Aβ_o_s and tautomycin together restored exocytosis to approximately control levels (Figure S3B). This would be consistent with a scheme in which Ca_V_2.1 function is regulated by a tonic cycle of phosphorylation and dephosphorylation, with Aβ_o_s and tautomycin acting to inhibit the relevant kinase and phosphatase, respectively. However, alternative interpretations are also possible, and further study will be required to establish the identity of the Ca_V_2.1 phosphatase beyond doubt.

### Aβ_o_s bind presynaptic α7-nAChR to drive ENaC membrane insertion and potentiate exocytosis

To complete the pathway linking Aβ_o_s to Ca_V_2.1, we sought to connect Aβ_o_s to the mobilization and membrane insertion of ENaCs, a process that is generally poorly understood, although it is known to be Ca^2+^ dependent.^36^ While the Ca^2+^ source driving ENaC insertion in neurons is not known, nicotinic acetylcholine receptors containing the Ca^2+^-permeable α7 subunit (α7-nAChRs) are strong candidates, since they have been reported to bind Aβ peptides with picomolar affinity and are expressed on hippocampal presynaptic boutons, where they can be directly activated by Aβ_o_s to produce a Ca^2+^ rise.^37^ To study the binding of Aβ_o_s to presynaptic α7-nAChR, we used CRISPR-Cas9 gene editing to knock out α7-nAChR expression in cultured hippocampal neurons, confirming the efficacy of the CRISPR-Cas9 construct in abolishing expression of the protein at the cell surface with a fluorescently tagged high-affinity ligand, α-bungarotoxin CF594 (Figure 3E). We showed that axons and boutons of neurons lacking surface α7-nAChR bound significantly fewer Aβ_o_s conjugated with the fluorophore tetramethyl rhodamine (TAMRA) (Figures 3E and 3F), a tag that has previously been shown to have no effect on the proportions of different Aβ assembly states present in Aβ_o_ preparations made according to our protocol.^38^ Knockout of α7-nAChR did not significantly alter the postsynaptic/dendritic binding of Aβ_o_s (Figure S3C), suggesting that their predominant postsynaptic binding partners are likely to be proteins other than α7-nAChR. We then used immunofluorescence to show that α-bungarotoxin treatment of Aβ_o_-incubated neurons prevents the insertion of ENaCs into the presynaptic cell membrane (Figure S3D), confirming that Aβ_o_-mediated activation of α7-nAChR lies mechanistically upstream of ENaC recruitment. We also used α-bungarotoxin to test whether α7-nAChR function is necessary for the ability of Aβ_o_s to enhance exocytosis and found that, while α-bungarotoxin treatment alone did not alter exocytosis in response to a 10 Hz stimulus train, it completely prevented the effects of Aβ_o_s (Figure 3G). Thus, an initial nAChR-mediated Ca^2+^ rise can be converted, via the recruitment of ENaCs, to a chronic, depolarization-driven cytoplasmic [Ca^2+^] elevation that ensures efficient activation of the Ca^2+^ sensor in order to sustain a chronic effect on exocytosis.

Together, our data support a model of presynaptic Aβ_o_ signaling that functions as follows. Aβ_o_s elicit presynaptic Ca^2+^ entry via α7-nAChR to drive the insertion of an intracellular pool of ENaCs into the presynaptic membrane. ENaCs mediate a chronic depolarization that, by activating Ca_V_2.3, elevates basal cytoplasmic [Ca^2+^], in turn activating PKC. This kinase then phosphorylates and inactivates the negative Ca_V_2.1 regulator GSK-3β, thereby potentiating Ca_V_2.1 function and neurotransmitter release (Figure 3H).

### Probability of release is enhanced in a Ca_V_2.1-dependent manner at CA3-CA1 synapses in Aβ_o_-treated hippocampal slices

**Figure 4:**
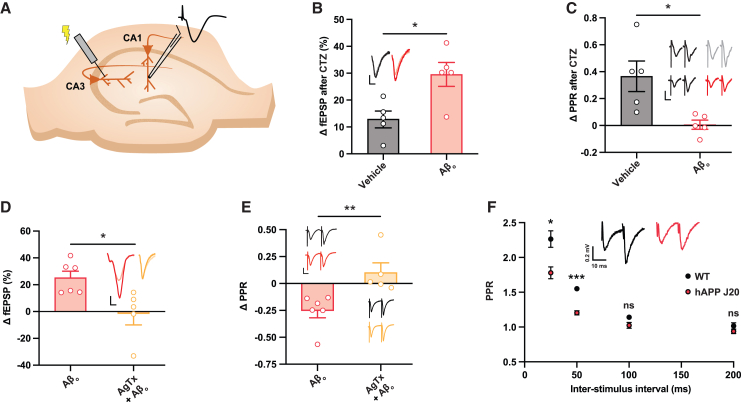
Probability of release is enhanced in a Ca_V_2.1-dependent manner at CA3-CA1 synapses in Aβ_o_-treated hippocampal slices (A) Schematic showing experimental setup for recording of field EPSPs evoked in CA1 of acute hippocampal slices by stimulation of the Schaffer collateral (CA3-CA1) pathway. (B) Acute hippocampal slices were perfused with buffer containing vehicle or Aβ_o_s, and stable basal synaptic transmission was recorded from CA1 for at least 10 min before addition of cyclothiazide (CTZ; 100 μm). Graph shows the percentage change in field EPSP (fEPSP) 10 min after CTZ addition (vehicle, *n* = 5 slices from two mice, and Aβ_o_, *n* = 5 slices from three mice). Inset shows sample traces before (faint lines) and after (bold lines) CTZ addition. Scale bars, 0.5 mV and 5 ms. (C) Change in paired-pulse ratio (PPR) measured in acute slices described in (B). Sample traces are responses before (black) and after (gray/red) addition of CTZ. Scale bars, 1 mV and 5 ms. (D) Stable recordings were established from acute hippocampal slices perfused with buffer containing CTZ, to prevent Aβ_o_-induced AMPAR desensitization, either with or without ω-agatoxin IVA. Graph shows the percentage change in fEPSP 30 min after addition of Aβ_o_s (10 nM) (Aβ_o_, *n* = 6 slices from six mice, and Aβ_o_ + ω-agatoxin IVA, *n* = 5 slices from five mice). Inset shows sample traces before (faint lines) and after (bold lines) Aβ_o_ addition. Scale bars, 0.5 mV and 5 ms. (E) Change in PPR measured in acute slices described in (D). Sample traces are responses before (black) and 30 min after (red/yellow) addition of Aβ_o_s. Scale bars, 1 mV and 5 ms. (F) PPR at CA3-CA1 synapses assessed at various interstimulus intervals in acute hippocampal slices from 4- to 8-month-old hAPP J20 and littermate wild-type control mice. CTZ was again added to prevent Aβ_o_-induced AMPAR desensitization (wild type and hAPP both *n* = 6 slices from three mice). Representative traces show responses at 50 ms intervals. Scale bars, 0.2 mV and 10 ms. Two-way ANOVA with *post hoc* t test and Sidak correction. Error bars represent ± SEM. ^∗^*p* < 0.05, ^∗∗^*p* < 0.01, ^∗∗∗^*p* < 0.0001, and ns, non-significant.

To support the pathophysiological relevance of these neuronal culture-based findings, we wished to explore a potential role for Ca_V_2.1 activity in AD-associated synaptic phenotypes in a more intact setting. We turned initially to electrophysiological recordings in Aβ_o_-incubated acute (*ex vivo*) hippocampal slices from 7- to 8-week-old mice (Figure 4A). We used 10 nM Aβ_o_ for these experiments, which has an essentially identical effect to the higher concentration used in culture work (Figures S2A and S2B). The paired-pulse ratio (PPR), which is the ratio of response amplitudes to each of a pair of closely spaced stimuli, is an electrophysiological index of the probability of release.^39^ We first assessed PPR at CA3-CA1 (Schaffer collateral) synapses and found no apparent effect of Aβ_o_ incubation (Figure S4A). However, Aβ_o_s have been shown to enhance basal desensitization of synaptic α-amino-3-hydroxy-5-methyl-4-isoxazolepropionic acid receptors (AMPAR),^40^ and changes in AMPAR desensitization can profoundly affect measurements of synaptic transmission, and in particular PPR, at CA3-CA1 synapses.^40^^,^^41^^,^^42^ Therefore, to understand whether AMPAR desensitization influences measurements of PPR in the presence of Aβ_o_s, we added the AMPAR desensitization inhibitor cyclothiazide while recording from slices either with or without Aβ_o_s in the perfusing ACSF. We found that, while the drug caused a slight enhancement in excitatory postsynaptic potential (EPSP) and a significant increase in PPR in control slices, there was a greater enhancement in EPSP but no change in PPR in Aβ_o_-perfused slices (Figures 4B and 4C). These observations would be in keeping with a role for AMPAR desensitization in PPR under control conditions, limiting the size of the response to the second pulse relative to the first as suggested by previous work.^41^ Following exposure to Aβ_o_s, however, our data suggest that basal desensitization is enhanced, essentially abolishing this effect.

We used cyclothiazide to study the effect of Aβ_o_s on PPR free of desensitization-related confounds. We found that, under these conditions, exposure to Aβ_o_s did result in a significant reduction in PPR, indicating an enhanced release probability (Figure S4B). Consistent with this, we have previously shown a similar effect of Aβ_o_s in hippocampal slices using direct presynaptic imaging.^43^ We then asked whether this effect was dependent upon Ca_V_2.1. In the presence of cyclothiazide, CA3-CA1 field EPSPs were enhanced and PPR was again diminished by the addition of Aβ_o_s, and these effects were robustly prevented by Ca_V_2.1 blockade with ω-agatoxin IVA (Figures 4D and 4E).

To explore Aβ-mediated alterations in synaptic function *in vivo*, we used an established and well-characterized model mouse line, J20, which carries a human APP (hAPP) transgene bearing two pathogenic mutations.^44^ We first sought to confirm whether hippocampal neurons in 4- to 8-month-old hAPP transgenic mice show evidence of elevated neurotransmitter release by measuring PPR at CA3-CA1 synapses in acute hippocampal slices, this time using a variety of interpulse intervals to increase the sensitivity of the study. As previously, we included cyclothiazide in these experiments to prevent confounding effects due to AMPA receptor desensitization. We found that PPR at 25 and 50 ms intervals was significantly reduced in hAPP mice compared to wild-type littermate controls, indicating enhanced probability of release (Figure 4F).

### Ca_V_2.1-dependent enhancement of synaptic vesicle exocytosis at CA3-CA1 synapses in hAPP J20 mice

**Figure 5:**
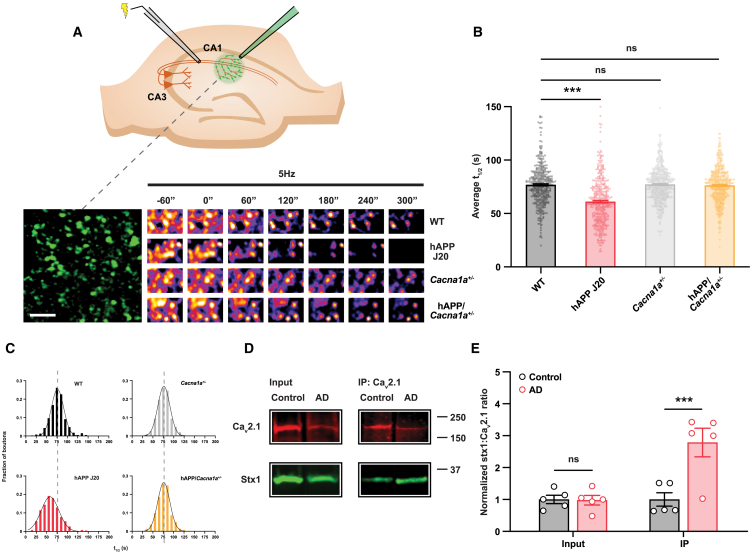
Ca_V_2.1-dependent enhancement of synaptic vesicle exocytosis at CA3-CA1 synapses in hAPP J20 mice (A) Schematic illustrating experimental protocol for visualizing synaptic vesicle exocytosis at CA3-CA1 synapses with FM 1-43 dye. Electrical stimulation of CA3 axons (10 Hz/120 s) turns over the total recycling pool of synaptic vesicles while the dye is applied to CA1, where it is taken up by vesicles as they are endocytosed. After washing to remove bound extracellular FM 1-43, the same axons are stimulated at 5 Hz to unload the dye. Bottom left is a representative field of dye-labeled presynaptic terminals in CA1 from wild-type hippocampus. Bottom right: sample time-lapse images demonstrating stimulus-driven dye loss at synaptic puncta in different mouse genotypes as indicated. Scale bar (applies to all images), 5 μm. (B) Average half-life (t_1/2_) of fluorescence unloading for individual puncta fitted with first-order exponential decay functions (wild type, *n* = 394 puncta from five slices; hAPP, *n* = 401 puncta from five slices; *Cacna1a*^+/−^, *n* = 542 puncta from six slices; and hAPP/*Cacna1a*^+/−^, *n* = 557 puncta from six slices). ANOVA with *post hoc* t test and Dunnett’s correction. (C) Relative frequency distribution histograms of t_1/2_ for each genotype with superimposed fitted Gaussian distribution (black line). Dashed lines represent mean t_1/2_ for wild-type slices. (D) The Ca_V_2.1 α_1A_ subunit was immunoprecipitated from purified synaptosomal membrane fractions prepared from the frontal cortex (Brodmann area 9) of AD patients and age-matched controls. Following SDS-PAGE separation, samples were probed with Ca_V_2.1 and syntaxin 1 (Stx1) antibodies as indicated. Representative immunoblot bands from both input samples and immunoprecipitates are shown. (E) Raw immunoblot Stx1:Ca_V_2.1 signal ratios from each individual were normalized to the control group mean. Enhanced Stx1:Ca_V_2.1 ratio in immunoprecipitated samples indicates stronger interaction between Ca_V_2.1 and syntaxin 1 in the brains of AD patients (both groups *n* = 5). Repeated measures ANOVA with *post hoc* t test and Sidak correction. Error bars represent ± SEM. ^∗∗∗^*p* < 0.0001 and ns, non-significant.

To further circumvent any potential confounding effects of postsynaptic changes that could influence our measurements, we used the styryl dye FM 1-43 to image synaptic vesicle exocytosis directly in native hippocampal tissue.^45^ Acute hippocampal slices were prepared from 4- to 8-month-old hAPP transgenic mice, and CA3 axons were stimulated while the dye was applied to CA1 to label the total recycling pool of synaptic vesicles (Figure 5A). A subsequent 5 Hz stimulus train revealed significantly faster dye unloading in hAPP vs. littermate control slices (Figures 5B and 5C). These results corroborate the electrophysiological data indicating enhanced neurotransmitter release in hAPP transgenic mice. To gain insight into the mechanism underlying enhanced release in hAPP transgenic mouse slices, we crossed the J20 line with transgenic mice bearing a heterozygous deletion of the gene encoding the pore-forming α1 subunit of Ca_V_2.1 VGCC (*Cacna1a*^+/−^).^46^ While homozygous *Cacna1a* ablation results in severe ataxia, seizures, and premature mortality,^46^ mice carrying a heterozygous deletion lack any readily apparent phenotype.^47^ However, synaptic transmission in *Cacna1a*^+/−^ slices shows a reduced dependence on Ca_V_2.1 (Figure S4C), and we therefore hypothesized that if enhanced Ca_V_2.1 activity mediates the effects of Aβ_o_s on neurotransmitter release, these effects would be ameliorated by the *Cacna1a*^+/−^ genotype. We first excluded a background effect of the *Cacna1a*^+/−^ genotype by confirming that FM dye unloading is not significantly different from that seen at wild-type synapses (Figures 5B and 5C). We then showed that the partial genetic suppression of Ca_V_2.1 was sufficient to normalize neurotransmitter release at hAPP synapses (Figures 5B and 5C), consistent with a critical mechanistic role for Ca_V_2.1 in the enhancement of release in AD model mice *in vivo*. To support the relevance of these experiments to the earlier acute slice work, we also confirmed that the *Cacna1a*^+/−^ genotype conferred resistance to the effects of Aβ_o_ incubation on presynaptic function as measured using PPR (Figure S4D).

### Evidence of enhanced Ca_V_2.1 function in hAPP mouse and human AD brains

Finally, we asked whether Ca_V_2.1 function is enhanced in human AD brains. Because it is not possible to assess this directly in postmortem material, we instead took advantage of an indirect index of Ca_V_2.1 activation, namely the tighter physical coupling of Ca_V_2.1 to the neurotransmitter release machinery that is part of the upregulation of Ca_V_2.1 function by Aβ_o_s (Figures S2F and S2G) and of Ca_V_2.1 regulation by GSK-3β more generally.^32^ The strength of Ca_V_2.1 binding to syntaxin 1, a SNARE protein that mediates vesicular neurotransmitter release, provides a measure of this coupling that can be assessed by co-immunoprecipitation. Accordingly, Ca_V_2.1 was immunoprecipitated from cortical synaptosomal membrane samples prepared from a cohort of AD patients and non-demented control subjects (Table S1). Immunoprecipitates were subjected to electrophoresis and western blotting with antibodies against Ca_V_2.1 and syntaxin 1. This showed that Ca_V_2.1 binds more syntaxin 1 in AD brains than in those of control subjects (Figures 5D and 5E), indicating a stronger Ca_V_2.1-SNARE association. We corroborated this observation in aged hAPP mice, supporting their relevance to human disease (Figures S5A and S5B). We noted incidentally that our western blots showed an apparent global reduction in the expression of presynaptic proteins in AD patients (Figure 5D), which was expected, since extensive loss of presynaptic terminals is a feature of advanced AD.^48^

## Discussion

Dysregulation of synaptic transmission is an early and critical pathogenic event in AD. While plentiful evidence implicates small oligomers of the Aβ peptide in a causal role, the underlying mechanisms remain only partially understood. Here, we show that Aβ_o_s engage a presynaptic ENaC - Ca_V_2.3 - PKC - GSK-3β signal transduction pathway that specifically enhances presynaptic Ca_V_2.1 VGCC activity, potentiating action-potential-evoked synaptic vesicle exocytosis. We find evidence that the pathway is active in hAPP transgenic mouse models *in vivo* and human AD brains and demonstrate that either pharmacological Ca_V_2.1 inhibition or genetic Ca_V_2.1 haploinsufficiency is sufficient to normalize neurotransmitter release. These findings reveal a previously unrecognized mechanism driving synaptic dysfunction in AD and identify a number of potentially tractable targets for future therapeutic approaches aimed at the restoration of normal synaptic function. In addition, our data indicate that this mechanism drives excitatory neuronal hyperactivity in hippocampal neuronal networks in culture and is therefore likely, alongside other mechanisms previously described,^4^^,^^5^ to contribute to aberrantly enhanced corticohippocampal excitatory activity *in vivo*. While synaptic loss is the major substrate of late-stage cognitive decline in AD,^1^ aberrant network-level activity is thought to play a key role much earlier in the disease process.^3^^,^^49^ Therapeutic correction of presynaptic functional deficits might, therefore, show benefit from even prodromal stages of AD.

While postsynaptic deficits, such as impairment of synaptic plasticity, have long been recognized as a critical component of Aβ_o_-induced synaptic dysfunction and therefore cognitive decline,^1^ presynaptic deficits may be as significant. Indeed, it is well established that perturbations in neurotransmitter release are an important substrate of cognitive changes in a wide variety of neurological disorders.^50^ However, the relatively small body of existing work on presynaptic effects of Aβ_o_ demonstrates a striking diversity of sometimes contradictory effects and mechanisms.^8^^,^^26^^,^^51^^,^^52^^,^^53^^,^^54^ While it is certainly possible that Aβ_o_ exhibits pleiotropic actions at the presynaptic terminal, it may also be significant that these effects are reported in a wide range of largely *in vitro* experimental systems, with many studies reliant on a single one. Our study initially exploited the superior experimental and optical access of neuronal cultures to identify an effect as well as to gain detailed insight into the underlying mechanisms. However, an equally important aim was then to support both the effect and the key elements of the mechanism with data from more intact, pathophysiologically relevant systems, including hAPP transgenic mice and human tissue, which enhances confidence in our conclusions.

Another cornerstone of our approach was to use, where possible, high-resolution optical methodologies to assess presynaptic neurotransmitter release directly, rather than relying on indirect approaches such as electrophysiology. Not only does this avoid a variety of potential experimental confounds,^45^ here, it helped to uncover an important issue affecting the use of electrophysiological recordings of the PPR to evaluate neurotransmitter release probability. In the physiological context, PPR in part reflects AMPA receptor desensitization that is induced by the first of the paired stimuli, and without this it is artifactually increased^41^^,^^42^; our data appear to confirm this. However, in the presence of pathophysiologically relevant concentrations of Aβ_o_s, our data suggest that AMPA receptors are constitutively fully desensitized, so that first pulse-induced desensitization cannot take place. The result is an artifactual increase in PPR in the presence of Aβ_o_s, giving a falsely low estimate of release probability.^39^ Accordingly, use of the AMPA receptor desensitization inhibitor cyclothiazide can unmask the effect of Aβ_o_s on PPR, which is otherwise hidden. PPR has been widely used within the AD field for many years, very often to exclude Aβ-induced presynaptic effects.^5^^,^^8^^,^^40^^,^^55^ However, PPR data obtained in the presence of elevated concentrations of Aβ should be interpreted with this critical issue borne in mind.

The specific signaling pathway that we identify linking Aβ_o_s to enhanced Ca_V_2.1 activity places some components in novel or non-canonical functional roles. While *in vivo* regulation of Ca_V_2.1 by a variety of presynaptic interactors, including CaM, G-protein-coupled receptors, and SNAREs, is well recognized,^12^ regulation via GSK-3β-mediated phosphorylation of the synprint site has only been described as a consequence of non-physiological manipulations *in vitro*^32^ and has not previously been implicated in a specific physiological or pathological pathway. Here, we begin to fill this knowledge gap by demonstrating a role for GSK-3β regulation of Ca_V_2.1 in Aβ_o_-mediated synaptic dysfunction. Further upstream in the signaling pathway, we implicate Ca^2+^ entry via Ca_V_2.3, itself opened by ENaC-mediated depolarization, in the activation of PKC, which then phosphorylates and negatively regulates GSK-3β. The distinct molecular properties of Ca_V_2.3, which opens at significantly more negative potentials than the principal presynaptic VGCC subtypes Ca_V_2.1 and Ca_V_2.2, suggest that it may be particularly amenable to activation by modest, subthreshold depolarization^23^ such as that mediated via ENaCs at the presynaptic terminal.^21^ Presynaptic Ca_V_2.3 has previously been implicated mainly as a trigger of spontaneous neurotransmitter release, a role that also exploits its greater probability of opening at close to resting membrane potential.^56^ Here, however, its special properties allow it to function in the novel context of an intracellular signal transduction pathway, where it serves to link subthreshold membrane depolarization to the upregulation of kinase activity.

In summary, our data demonstrate a critical role for enhanced Ca_V_2.1 activity in Aβ_o_-mediated presynaptic dysfunction. Furthermore, they identify Ca_V_2.1, together with components of the signaling pathway linking Aβ_o_s to Ca_V_2.1, as a potential target that may merit further investigation for therapeutic approaches aimed at mitigating the toxicity of Aβ_o_s at either the synapse or the network level via the normalization of presynaptic function.

### Limitations of the study

This study has some technical limitations. The neuronal culture work, as well as some of the acute slice work, is reliant on the use of a variety of pharmacological inhibitors of protein function. While these are valuable tools, pharmacological agents are less specific than genetic interventions and come with off-target effects of varying severity and significance. While the agents used in our study have relatively high target specificity, particularly the peptides such as the Ca_V_2.1 blocker ω-agatoxin IVA, contamination of responses by off-target effects is always a possibility. In our case, we have attempted to mitigate this as much as possible by using these drugs for as short a duration, and at as low a concentration, as possible. We have also supported each pharmacological experiment with at least one orthogonal line of evidence using a non-pharmacological approach. A further potential limitation of the study relates to the use of an hAPP transgenic mouse model. While these mice represent the most pathophysiologically intact model available for an interventional study of this kind, they do not represent a complete model of the human AD brain, since they lack key features, including the presence of pathological tau deposits and, perhaps most importantly, the many and varied effects of decades of brain aging on which the disease process is usually superimposed. In studies using these mice, it is therefore highly desirable to obtain some evidence from human AD brain tissue in support of the main conclusions, as we have done here.

## Resource availability

### Lead contact

Requests for further information and resources and reagents should be directed to and will be fulfilled by the lead contact, Alexander Jeans (alexander.jeans@pharm.ox.ac.uk).

### Materials availability

This study did not generate new unique reagents.

### Data code and availability

• All data supporting the findings of this study are available either within the paper or from the lead contact upon request.
• This paper does not report original code.
• Any additional information required to reanalyze the data reported in this paper is available from the lead contact upon request.

## Acknowledgments

We thank Leon Lagnado and Yongling Zhu for gifts of plasmids and the members of the Emptage laboratory for comments on the manuscript. This work was supported by an MRC (UK) Clinician Scientist Fellowship (G0802812) and Centenary Award to A.F.J.

## Author contributions

A.F.J. designed experiments, performed experiments, analyzed data, and wrote the paper. Z.P., H.C., W.F., S.A., and S.D. performed experiments and analyzed data. W.L.K. characterized and contributed Aβ_o_ preparations. A.M.J.M.v.d.M. contributed the *Cacna1a*-knockout mouse line. N.J.E. provided oversight for the work. All authors revised the manuscript.

## Declaration of interests

The authors declare no competing interests.

## STAR★Methods

### Key resources table

**Table:** 

REAGENT or RESOURCE	SOURCE	IDENTIFIER
**Antibodies**		

Guinea pig polyclonal anti-synaptophysin	Synaptic Systems	Cat# 101 004; RRID AB_1210382
Mouse monoclonal anti-GSK-3β	Cell Signaling Technology	Cat# 9832; RRID AB_10839406
Rabbit polyclonal anti-phospho-GSK-3β (S9)	St. John’s Laboratory	Cat# STJ22160
Rabbit polyclonal anti-ENaC α-subunit	StressMarq	Cat# SPC-403; RRID AB_10640131
Goat polyclonal anti-guinea pig AlexaFluor 405-conjugated	Abcam	Cat# ab175678; RRID AB_2827755
Goat polyclonal anti-mouse AlexaFluor 488-conjugated	Abcam	Cat# ab150117; RRID AB_2688012
Goat polyclonal anti-rabbit AlexaFluor 594-conjugated	Abcam	Cat# ab150080; RRID AB_2650602
Rabbit polyclonal anti-CACNA1A (Ca_V_2.1)	Alomone Labs	Cat# ACC-001; RRID AB_2039764
Mouse monoclonal anti-syntaxin 1	Synaptic Systems	Cat# 110 011; RRID AB_887844
Goat polyclonal anti-mouse IRDye 800CW-conjugated	Li-Cor Biosciences	P/N 926-32210; RRID AB_621842
Donkey polyclonal anti-rabbit IRDye 680LT-conjugated	Li-Cor Biosciences	P/N 926-68023; RRID AB_10706167

**Bacterial and virus strains**		

Adenovirus Ad-SypHyA4	Vector Biolabs	N/A

**Biological samples**		

Alzheimer’s disease and control BA9 brain tissue	London Neurodegenerative Diseases Brain Bank (https://www.kcl.ac.uk/neuroscience/facilities/brain-bank)	N/A

**Chemicals, peptides, and recombinant proteins**		

Lipofectamine 2000	Thermo Fisher Scientific	Cat# 11668027
Aβ_1-42_ peptide	Abcam	Cat# ab82795
NBQX	Abcam	Cat# ab144489
D-AP5 (APV)	Abcam	Cat# ab120003
ω-Agatoxin IVA	Alomone Labs	Cat# STA-500
ω-Conotoxin GVIA	Alomone Labs	Cat# C-300
Amiloride	Sigma-Aldrich	Cat# A7410
BAPTA	Thermo Fisher Scientific	Cat# B1204
SNX-482	Alomone Labs	Cat# RTS-500
SIB-1757	Tocris	Cat# 1215
Go 6976	Abcam	Cat# ab141413
Tautomycin	Sigma-Aldrich	Cat# 580551
α-Bungarotoxin	Sigma-Aldrich	Cat# 203980
Ionomycin	Thermo Fisher Scientific	Cat# I24222
Syn-PER	Thermo Fisher Scientific	Cat# 87793
TAMRA-conjugated Aβ_1-42_	AnaSpec	Cat# ANA60476
α-Bungarotoxin CF594	Biotium	Cat# BT00007
Cyclothiazide	Abcam	Cat# ab120323
FM1-43	Thermo Fisher Scientific	Cat# T3163
ADVASEP-7	Biotium	Cat# BT70029

**Critical commercial assays**		

Bradford Plus Protein Assay	Thermo Fisher Scientific	Cat# 23238
PKC kinase activity kit	Enzo Life Sciences	Cat# ADI-EKS-420A

**Experimental models: Organisms/strains**		

Wistar rats	Charles River Laboratories UK	Strain code 003; RRID RGD_737929
C57BL/6J mice	Oxford University Biomedical Services	RRID IMSR_JAX:000664
B6.Cg-*Zbtb20*^*Tg(PDGFB-APPSwInd)20Lms*^/2Mmjax (J20)	Mucke et al.^44^	https://www.jax.org/strain/006293#
*Cacna1a* knockout mice	Kaja et al.^46^	N/A
B6.Cg-Tg(tetO-APPSwInd)102Dbo/Mmjax	Jankowsky et al.^57^	https://www.jax.org/strain/007051

**Recombinant DNA**		

Plasmid SypHy	Granseth et al.^9^	Addgene plasmid# 24478
Plasmid SypH 2x	Zhu et al.^11^	Addgene plasmid# 37004
Plasmid SyGCamP5	Akerboom et al.^13^	N/A
Plasmid GAD67^pro^-vGAT-pH	Bae et al.^10^	N/A
Plasmid sgRNA *Chrna7* (g13)	This paper	N/A
Plasmid sgRNA luciferase	This paper	N/A

**Software and algorithms**		

iQ	Andor	https://andor.oxinst.com/products/; RRID SCR_014461
ImageJ	NIH	https://imagej.nih.gov/ij/; RRID SCR_003070
WinWCP	Strathclyde University	https://spider.science.strath.ac.uk/sipbs/software_ses.htm; RRID SCR_014713
Li-Cor Image Studio Lite	Li-Cor Biosciences	https://www.licor.com/bio/image-studio-lite/; RRID SCR_013715
MATLAB	MathWorks	https://www.mathworks.com/products/matlab.html; RRID SCR_001622
Prism	GraphPad	https://www.graphpad.com/scientific-software/prism/; RRID SCR_002798

### Experimental model and study participant details

#### Animal models

All mouse work was carried out in accordance with the Animals (Scientific Procedures) Act, 1986 (UK) and under project and personal licenses approved by the Home Office (UK). Both the *Cacna1a* knockout (from Prof. A. van den Maagdenberg, Leiden University Medical Centre) and J20 hAPP transgenic (kind gift of Prof. D. Anthony, University of Oxford) mouse lines were on a C57BL/6J background, and the lines were maintained by crossing heterozygotes or hemizygotes with wild-type C57BL/6J mice. Crossing hAPP hemizygote with *Cacna1a*^*+/-*^ mice yielded all four of the genotypes used in the FM dye assays, and animals used in these experiments were therefore littermates. For co-immunoprecipitation experiments only, another mouse line carrying, like J20, an APP transgene with Swedish and Indiana mutations, was used. Line B6.Cg-Tg(tetO-APPSwInd)102Dbo/Mmjax was crossed with a CamKIIα-tTA line to activate hAPP expression,^57^ and 20 month old double transgenic (tTA/hAPP) individuals along with accompanying non-transgenic littermate controls, a kind gift of Dr. Mariana Vargas-Caballero, were used. Mice were housed in group cages in a facility with controlled temperature and lighting (alternating 12 hour light/dark cycles). All genotyping was carried out by Transnetyx Inc. Mice of both sexes were used in all experiments.

#### Primary cultures

Dissociated hippocampal cultures were prepared from E18 Wistar rat embryos of both sexes as already described.^18^ Briefly, hippocampal neurons were seeded onto poly-D-lysine-coated coverslips and cultured in Neurobasal medium supplemented with 2% fetal calf serum (FCS), 2% B27, 1% Glutamax and 1% penicillin/streptomycin. The day after plating, half the medium was changed for Neurobasal supplemented with 2% B27 and 1% Glutamax only; this medium was used for all further feeds. Cultures were maintained in a humidified incubator at 37°C with 5% CO_2_.

### Method details

#### Preparation, characterization and use of Aβ oligomers

Oligomers were prepared from Aβ_1-42_ peptide as previously described.^58^ Briefly, solid Aβ_1-42_ was dissolved in cold hexafluoro-2-propanol (HFIP; Sigma-Aldrich). The peptide was incubated at room temperature for at least 1 hour to establish monomerization and randomization of structure. The HFIP was aliquoted and allowed to evaporate overnight, followed by 10 minutes in a Savant Speed Vac. The resulting peptide was stored as a film at -80 °C. The film was dissolved in anhydrous dimethylsulfoxide (Sigma-Aldrich) to 5 mM, diluted to 100 μM with Ham’s F12 (without phenol red, with glutamine; Caisson Laboratories) and briefly vortexed. The solution was incubated at 4 °C for 22-24 hours and soluble oligomers obtained by centrifugation at 14,000 g for 10 minutes at 4 °C. Protein concentration was estimated using the Bradford Plus Protein Assay and a bovine serum albumin (BSA) standard. For shipping purposes, small aliquots of the soluble oligomers were dried using a Savant Speed Vac and reconstituted with cold sterile water and gentle pipetting immediately prior to use. Tetramethyl rhodamine (TAMRA)-conjugated Aβ_1-42_ was subjected to the same protocol to prepare fluorescently-tagged oligomers.

#### Adenoviral infection and plasmid transfections

SypHy (kind gift of Prof. L. Lagnado) was cloned into an adenoviral expression vector using the Ad-HQ system, and packaged to produce an active human Adenovirus Type 5 (dE1/E3). This was used to infect cultured hippocampal neurons 8 days after plating at a multiplicity of infection (MOI) of 0.2. All other plasmids were introduced into neurons 8 days after plating using Lipofectamine 2000. For each well, DNA and Lipofectamine were added to 200 μL Neurobasal at a ratio of 3 μg:3 μL and incubated for 20 minutes before transfection, which was carried out in 2 mL of medium per well. Transfection mix was incubated with the cultures for 1 hour before being removed and replaced with a 2:1 ratio of conditioned to fresh medium. The SypH 2x plasmid was a gift of Dr. Y. Zhu, the GAD67^pro^-vGAT-pH plasmid was a gift of Prof. S. H. Kim and SyGCamP5 was a gift of Prof. L. Lagnado. The CRISPR/Cas9 plasmids were generated by the Weatherall Institute of Molecular Medicine Genome Engineering Facility (University of Oxford) and based on the vector px458 into which sequences coding for sgRNA directed against either the firefly luciferase gene (control) or mouse *Chrna7* (g13)^59^ were cloned.

#### Patch-clamp electrophysiology in cultured hippocampal neurons

Whole-cell patch recordings were made using either an Axoclamp 900A or an Axoscope 2B amplifier (both Axon Instruments). Data was low-pass filtered at 3k Hz, sampled at >10kHz. Data was acquired using WinWCP and analyzed using Clampfit software. Patch electrodes (4-8 MΩ) contained (in mM): 120 CsMeSO_4_, 10 KCl, 10 NaPhosphocreatine, 10 HEPES, 4 MgATP and 0.4 Na_3_GTP. Spontaneous activity was recorded in Tyrode’s solution containing (in mM): 120 NaCl, 30 Glucose, 25 HEPES, 5.4 KCl, 1.5 CaCl_2_ and 0.5 MgCl_2_.

#### Live cell imaging and analysis

Experiments were performed 14-21 days after plating (6-13 days after transfection) when synapses are mature. Coverslips were mounted in a Chamlide EC-B18 stimulation chamber (Live Cell Instrument) on the stage of an Olympus IX-71 inverted microscope fitted with a 100X, NA 1.40 UPlanSApo objective and an Andor iXon EM CCD camera, and stimulation and imaging of live neurons was carried out as described.^18^ Phluorin imaging was carried out at 1 Hz for 100 AP stimulation and 5 Hz for 1 AP stimulation; Ca^2+^ imaging was carried out at 10 Hz. All time series images were acquired with 2 X 2 pixel binning. Unless otherwise specified, Aβ_o_ were applied at 200 nM (monomer equivalent) in culture medium in the incubator for 2 hours. Where cells had been incubated with Aβ_o_ or vehicle, these were present throughout the experiment. Where the following were used they were added 10 minutes before Aβ_o_ or vehicle treatment and were present throughout the incubation and experiment: ω-Agatoxin IVA (100 nM); ω-conotoxin GVIA (250 nM); amiloride (100 μM); BAPTA (5 mM tetrapotassium salt); SNX-482 (500 nM); SIB-1757 (3 μM); Go 6976 (100 nM); tautomycin (2 nM); α-bungarotoxin (100 nM). In experiments measuring basal Ca^2+^ concentration, ionomycin was used at 10 μM following initial image acquisition to elicit maximal Ca^2+^ entry; the maximal signal value obtained is used to normalize the basal Ca^2+^ signal measurements to account for differences in SyGCamP5 expression. In experiments imaging binding of either α-bungarotoxin CF594 (100 nM) or TAMRA-conjugated Aβ_o_ (200 nM), which were applied in the incubator for 10 minutes or 2 hours respectively, the image acquisition system described above was used but with a 900 ms exposure time and no pixel binning.

Time series images were analyzed in ImageJ (http://rsb.info.nih.gov/ij) using the Time Series Analyzer plugin (http://rsb.info.nih.gov/ij/plugins/time-series.html). All visible varicosities were selected for analysis with a 2 μm diameter ROI. Terminals were excluded from analysis in pHluorin experiments if their maximum response to 40 AP or 100 AP was less than 2 SD of baseline noise; for Ca^2+^ experiments, terminals were excluded if their peak response to 1 AP averaged over 5 trials was less than 2 SD of baseline noise, or of they did not show a response to ionomycin application. Data exported from ImageJ were background adjusted and pHluorin data were normalized to the peak signal obtained following NH_4_Cl application (mean value of plateau over 5 seconds). Ca^2+^ indicator data were normalized to either the basal, unstimulated signal or to the signal elicited by ionomycin. Peak fluorescence in all experiments was taken at the end of the stimulation period. For analysis of single images generated during Aβ_o_ binding experiments, segmentation was first carried out using inbuilt ImageJ functionality to generate a mask from a 50 μm section of axon or dendrite in the GFP channel, and this was then used to measure mean fluorescence in the red fluorophore channel with additional background adjustment. All analysis was performed using custom-written macros or scripts in Microsoft Excel or MATLAB.

#### Optical fluctuation analysis

Optical fluctuation analysis analyses trial-to-trial variation in Ca^2+^ transients through VGCC to determine whether changes in Ca^2+^ influx are due to changes in the number (N), open probability (p) or unitary channel currents (q) of functional channels.^14^ We used the experimentally derived inverse squared coefficient of variation (CV^-2^) of boutonal Ca^2+^ transients along with a published value for p at cultured hippocampal terminals,^56^ as unfortunately this parameter cannot be experimentally measured, to calculate the mean N at each terminal under control conditions (N = 19.5). We then extrapolated both N and p to values expected if the experimentally observed increase in mean Ca^2+^ transient size following Aβ_o_ treatment were solely a result of an increase in either of these parameters alone, and used these to calculate the CV^-2^ that would be expected after Aβ_o_ treatment in each instance. Note that in optical fluctuation analysis, changes in N or p (or some combination of these) are associated with a change in CV^-2^, while changes in q are not.

#### Immunofluorescence and quantification

Cultured neurons were subjected to relevant treatments before being washed twice with PBS; where used, Aβ_o_ were applied at 200 nM for 2 hours. The cells were then fixed in 4% paraformaldehyde for 15 minutes on ice. For all experiments except surface ENaC staining, coverslips were then treated with 0.1% saponin or Triton X-100 on ice for 15 minutes, before incubation in 10% FCS in PBS at room temperature for 30 minutes. Cells were then incubated with anti-synaptophysin antibody (1:1000) together with either anti-GSK-3β (1:1000) and anti-phospho-GSK-3β (S9) (1:500), or anti-ENaC α-subunit (1:1000). For surface ENaC staining, the procedure was identical except that the primary antibody incubations were carried out in succession, anti-ENaC antibody first, and the permeabilization step, if included, was carried out between the two. Following primary antibody incubation, coverslips were washed, incubated for 1 hour at room temperature with various AlexaFluor-conjugated secondary antibodies, all used at 1 in 400 dilution, washed again and mounted using ProLong Gold antifade (Thermo Fisher Scientific).

Images were collected using an Olympus Fluoview FV1000 confocal system with an Olympus IX-81 inverted microscope, and either a 100X, NA 1.40 UPlanSApo or a 60X, NA 1.35 UPlanSApo oil immersion objective. Images were acquired in Olympus Fluoview software and analyzed using ImageJ as described in the main text or caption. Image segmentation was carried out using inbuilt ImageJ functionality to generate a mask from the synaptophysin channel, and this was then used to measure mean fluorescence in other fluorophore channels as required. For all experiments, image acquisition parameters, as well as any image thresholding applied, were fixed within an experiment to allow for comparison between conditions.

#### Protein kinase C activity assay

Synaptosomes were prepared 14 days after plating from individual 35 mm culture wells of hippocampal neurons cultured as described above. Syn-PER reagent was used according to the manufacturer’s instructions, and each pellet representing synaptosomes from a single well was resuspended in 100 μl Syn-PER, before this was then divided into three parts that were exposed to either a vehicle, Aβ_o_ (200 nM) or Aβ_o_ + SNX-482 (500 nM) treatment for 2 hours before immediate freezing at -80 °C. Protein concentration in each sample was assessed with a BCA assay, and PKC activity in the samples was then assessed with an ELISA-based kit used according to manufacturer’s instructions. Based on preliminary experiments with a purified PKC standard included with the kit, a 0.1 μg total protein equivalent of each sample was loaded per assay well, as this amount gave results within the dynamic range of the assay, and all samples were assayed in duplicate.

#### Preparation of acute hippocampal slices and slice electrophysiology

Field excitatory postsynaptic potentials (fEPSPs) were recorded in 300 μm thick acute hippocampal slices prepared as described^60^ from 7 to 8 week old C57BL/6J mice, or from aged, genotyped mice as specified. Slices were placed in an interface recording chamber perfused with oxygenated ACSF (2 mM Ca^2+^, 1 mM Mg^2+^) at 1 to 2 mL/min, and a bipolar stimulating electrode (FHC Inc., Bowdoin, ME, USA) was placed in Schaffer collaterals to deliver test and conditioning stimuli. For most experiments, a borosilicate glass recording electrode filled with artificial cerebrospinal fluid was positioned in stratum radiatum of CA1. Test responses to stimuli delivered at 0.067 Hz were recorded for at least 10 minutes prior to beginning experiments to ensure stable responses. Field potentials were amplified using a Digitimer NeuroLog amplifier, filtered below 3 Hz and above 3 KHz and digitized with a BNC-2090A converter (National Instruments). Recording was carried out on WinWCP software and analyzed using the Clampfit program. Unless otherwise stated, slices were incubated in drug treatments for > 2 hours prior to the experiment and the drugs were maintained in the perfusing ACSF for the duration of the recording. Concentrations used in all experiments were as follows: ω-agatoxin IVA 400 nM; Aβ oligomers 10 nM; cyclothiazide 100 μm. Other than these, drugs were not included in the experiments in order to preserve intact neuronal circuits. The magnitude of fEPSPs was determined as the gradient of the rising slope to avoid population spike contamination. Paired-pulse ratios were obtained by delivering two stimuli at intervals as specified and expressed as fEPSP2/fEPSP1.

#### FM dye loading and unloading in acute hippocampal slices

Slices of 300 μm thickness prepared as above were transferred to a custom-made recording chamber mounted on an Olympus BX50WI microscope fitted with a BioRad Radiance 2000 confocal scanhead (BioRad/Zeiss) and were superfused at 35°C with oxygenated ACSF (2 mM Ca^2+^, 1 mM Mg^2+^) supplemented with 10 μM NBQX and 50 μM APV to block recurrent activity. A patch pipette was filled with a 20 μM solution of the styryl dye FM1-43 in ACSF and placed in stratum radiatum of CA1 at a depth of approximately 100 μm. The dye was pressure applied for 3 minutes using a custom-made picospritzer before a 10 Hz train of 1200 stimuli (100 μA) was delivered to Schaffer collaterals using a glass stimulating electrode (4-8 MΩ) filled with 150 mM NaCl placed within 70 μm of the dye-filled pipette; the stimulating electrode was under the control of WIN WCP software and a DS3 stimulation box (Digitimer). Pressure application of dye was maintained throughout the loading stimulus and for 2 minutes afterwards to ensure completion of endocytosis. Slices were then perfused continuously in fresh ACSF containing 0.2 mM ADVASEP-7 for 15-20 minutes to wash residual FM dye from extracellular membranes. Imaging of labeled terminals was performed using a 60X, NA 1.1 LUMFL N objective (Olympus), a 488 nm Argon laser for excitation and a 500 nm long-pass emission filter. Image stacks were acquired every 15 seconds throughout the unloading stimulus (3000 stimuli at 10 Hz). Each image stack comprised 6 images of 512 x 512 pixels acquired at 1 μm intervals in the *z*-axis, and a digital zoom of 3X and 2X Kalman averaging were applied. Images were acquired using Zeiss LaserSharp software and analyzed using ImageJ and custom-written scripts in MATLAB.

#### Human tissue

Samples of human frontal cortex (Brodmann area 9) were obtained from the MRC London Neurodegenerative Diseases Brain Bank, King’s College, London (part of the Brains for Dementia Research initiative) with full ethical approval; 5 AD cases, all BrainNet Europe stage VI or modified Braak stage VI, and 5 age-matched controls, all BrainNet Europe or Braak stage I or II, were used (see Table S1).

#### Co-immunoprecipitation

For co-immunoprecipitations, approximately 500 mg of either human or mouse brain tissue was homogenized using a Dounce tissue grinder into 2 mL buffer A containing (in mM): 320 sucrose, 5 Tris base, pH 7.4, 2 EDTA, on ice; note that all buffers used throughout the immunoprecipitations included HALT protease and phosphatase inhibitor cocktail (Thermo Fisher Scientific) at 1:100. After a 10 minute centrifugation at 750 rpm, the supernatant was removed and centrifuged at 17,000 rpm for 1 hour. This yielded a membrane pellet which was resuspended in 1 mL buffer A + 1% CHAPS, and 300 μL of this was rotated for 3 hours at 4°C with Novex magnetic Protein G Dynabeads (Life Technologies) to which anti-Ca_V_2.1 or antibody had previously been bound (see below). After incubation, the beads were retrieved by magnetic separation and washed twice in buffer B containing (in mM): 150 NaCl, 25 Tris base, pH 7.4, 2 EDTA, to which 0.5% BSA and 0.4% CHAPS had also been added. Beads were then washed once in buffer B alone before being resuspended in 100 μL 2X Laemmli sample buffer with 5% β–mercaptoethanol for Western blotting. For binding of antibodies to Protein G Dynabeads, the supplied buffer was removed from 50 μL of bead suspension, and beads were washed once in buffer A + 0.5% BSA + 0.4% CHAPS. 5μL of anti-Ca_V_2.1 antibody (as used for Western blotting) was then added, and the bead/antibody mixture was rotated for 10 minutes at room temperature before beads were washed twice in buffer A + 0.5% BSA.

#### Western blotting

Samples were dissolved in 2X Laemmli sample buffer with 5% β–mercaptoethanol, heated to 60°C for 3 minutes and run on a precast 4-20% gradient SDS-PAGE gel (Thermo Fisher Scientific). The separated samples were transferred to a nitrocellulose membrane (Bio-Rad) before blocking with 5% milk and 1% horse serum in TBS with 0.05% Tween-20 (TBST) and subsequent probing with a mixture of anti-Ca_V_2.1 (1:200) and anti-stx1 (1:1000) antibodies. After three TBST washes, bound antibodies were detected with IRDye 680LT donkey anti rabbit IgG (1:20,000) and IRDye 800CW goat anti-mouse IgG (1:15,000) fluorescent secondary antibodies, washed three times in TBST and imaged on a Li-Cor Odyssey system. Image analysis was performed in Li-Cor Image Studio Lite software.

### Quantification and statistical analysis

Statistical analysis was performed using GraphPad Prism software. Data were assessed for normality using the Shapiro-Wilk test, and analyzed using parametric or non-parametric tests accordingly. Unless otherwise stated, the two-tailed unpaired Student’s t test was used to determine the statistical significance of observed differences between various conditions. Where other tests were used, this is clearly stated in the caption of the appropriate figure. Figure captions also contain the details of tests conducted, including n for each test. P values greater than 0.05 were regarded as non-significant. For optical fluctuation analysis, we determined whether changes in N, p or q best described our data using the Bayesian Information Criterion (BIC), with a BIC difference of 10 or more providing strong evidence in favor of the model with the lowest BIC score.^61^

## Supplemental information

Document S1. Figures S1–S5 and Table S1

Document S2. Article plus supplemental information
