# Supplementary material for: CaV2.1 mediates presynaptic dysfunction induced by amyloid β oligomers
Source: Cell Rep. 2025 Mar 23;44(4):115451. doi: 10.1016/j.celrep.2025.115451 (PMC12799594; doi:10.1016/j.celrep.2025.115451)
Supplement: Document S1. Figures S1–S5 and Table S1 [file mmc1.pdf]

Cell Reports, Volume *44*

Supplemental information

Ca_V2.1 mediates presynaptic dysfunction induced by amyloid β oligomers

Alexander F. Jeans, Zahid Padamsey, Helen Collins, William Foster, Sally Allison, Steven Dierksmeier, William L. Klein, Arn M.J.M. van den Maagdenberg, and Nigel J. Emptage

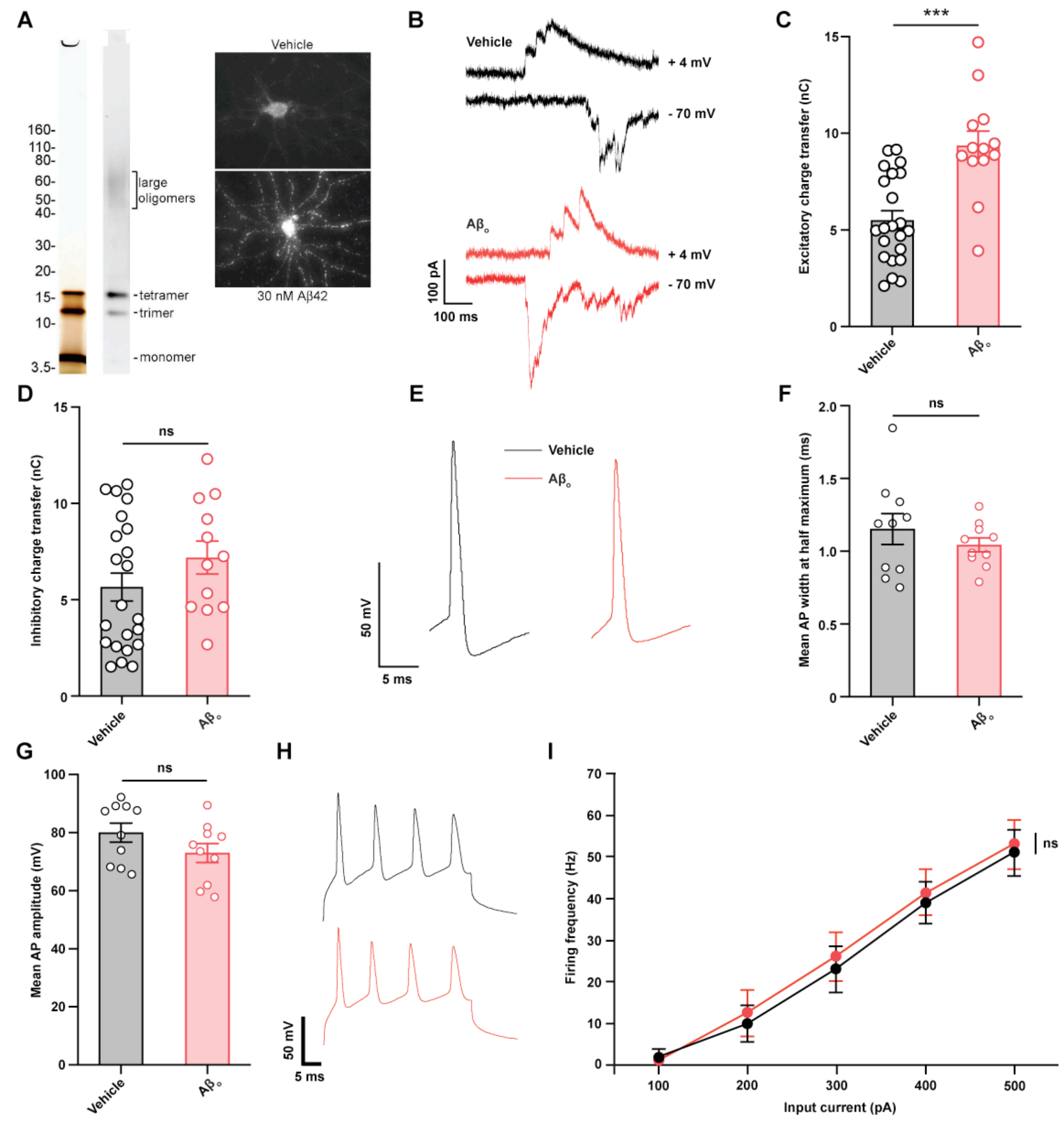

Figure S1. Related to Figure 1.

(A) Silver-stained SDS-PAGE gel of Aβ oligomers (left) shows a predominant monomer band in addition to trimers and tetramers. The smaller species are largely the result of the SDS-sensitivity of larger oligomers[S1]. Western blot immunostaining (middle) with oligomer-specific monoclonal antibody NU2 detected the presence of large SDS-stable oligomers in addition to trimers and tetramers. Mature (26 DIV) hippocampal cell cultures (right) incubated for 30 minutes at 37°C with oligomers formed at 30 nM $A\beta_{42}$ peptide shows specific synaptic binding detected by immunolabelling with oligomer-specific monoclonal antibody NU4.

(B) Effects of $A\beta_o$ on activity across neuronal populations. Sequential recordings of 150 second duration were made from neurons held in voltage clamp at -70 mV (the reversal potential for $GABA_A$ receptor-mediated currents) and +5 mV (the reversal potential for AMPA receptor-mediated currents) to isolate spontaneous EPSCs and IPSCs, respectively. Representative traces from cells treated as indicated.

(C) Average sEPSC (excitatory) charge transfer over the recording period in nC (control: n = 22 cells; $A\beta_o$: n = 13 cells).

(D) Average sIPSC (inhibitory) charge transfer over recording period in nC (control: n = 22 cells; $A\beta_o$: n = 12 cells).

(E) Representative traces of single depolarization-elicited action potentials from cells treated as indicated show no change in amplitude or duration following $A\beta_o$ treatment.

(F) Mean duration of action potentials measured as width at half maximum height (n = 10 cells).

(G) Mean amplitude of action potentials (n = 10 cells).

(H) Excitability is unchanged by $A\beta_o$: representative traces from cells treated as indicated showing trains of action potentials elicited by current injections (50 ms duration).

(I) Average frequency of action potentials elicited by varying injections of current (n = 10 cells). Two-way ANOVA. Note that membrane conductance (G_m) was unchanged in $A\beta_o$-treated cells (G_m in nS: control, 9.1 ± 2.9; $A\beta_o$, 12.3 ± 2.1. p>0.3).

Error bars represent ± s.e.m. $^{***}p < 0.0001$, ns = non-significant.

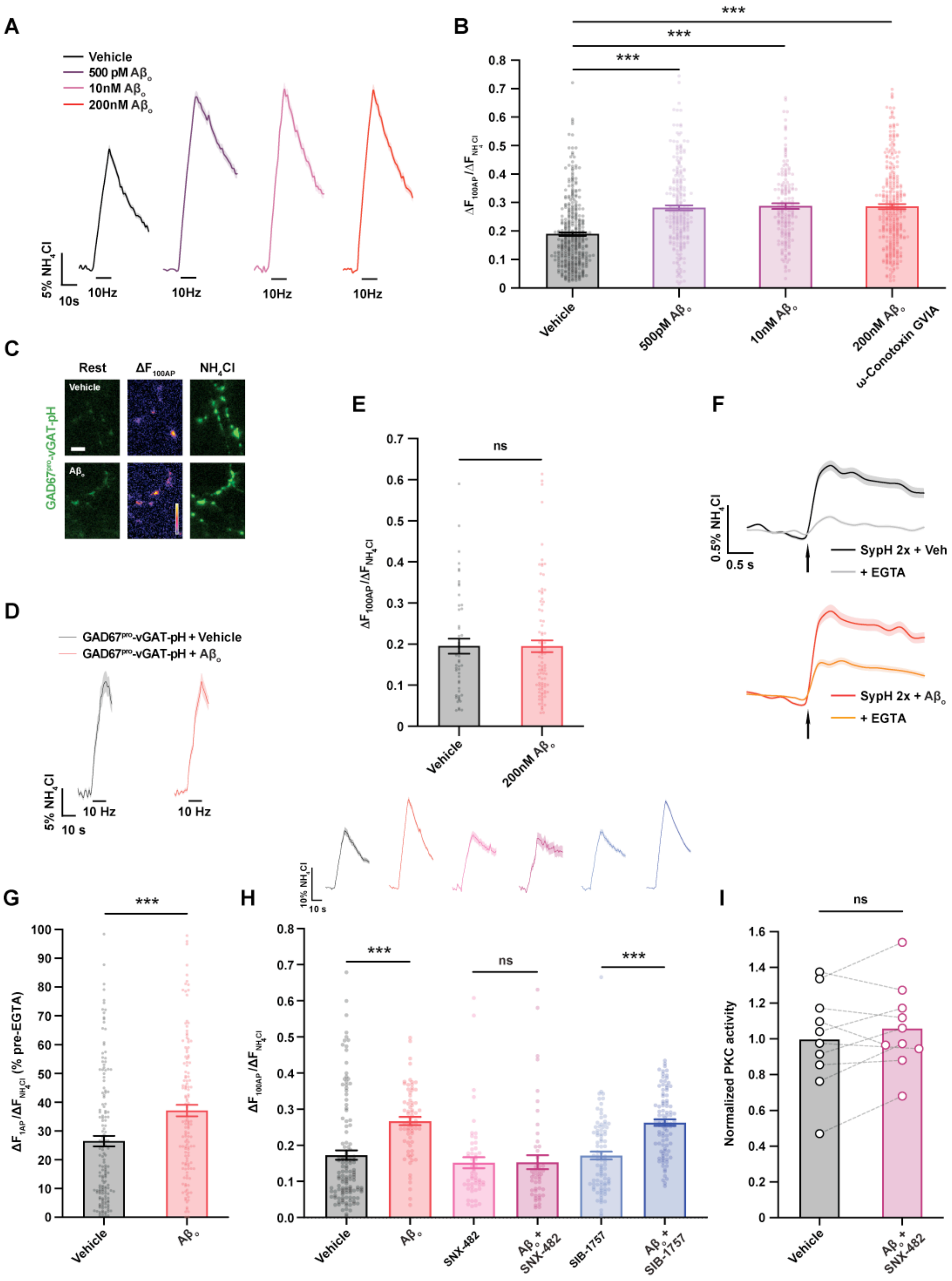

A
Vehicle
500 pM Aβo
10nM Aβo
200nM Aβo
5% NH4Cl
10s
10Hz
B
ΔF100AP/ΔFNH4Cl
***
Vehicle
500pM Aβo
10nM Aβo
200nM Aβo
ω-Conotoxin GVIA
C
Rest
ΔF100AP
NH4Cl
Vehicle
Aβo
GAD67pro-vGAT-pH
D
GAD67pro-vGAT-pH + Vehicle
GAD67pro-vGAT-pH + Aβo
5% NH4Cl
10 s
10 Hz
E
ns
ΔF100AP/ΔFNH4Cl
Vehicle
200nM Aβo
F
0.5% NH4Cl
0.5 s
SypH 2x + Veh
+ EGTA
SypH 2x + Aβo
+ EGTA
G
***
ΔF1AP/ΔFNH4Cl (% pre-EGTA)
Vehicle
Aβo
H
10% NH4Cl
10 s
***
ns
ΔF100AP/ΔFNH4Cl
Vehicle
Aβo
SNX-482
Aβo + SNX-482
SIB-1757
Aβo + SIB-1757
I
ns
Normalized PKC activity
Vehicle
Aβo + SNX-482

Figure S2. Related to Figures 1 & 2.

(A) Average SypHy fluorescence traces showing the response to 100 stimuli delivered at 10 Hz following incubation in $A\beta_o$ concentrations as indicated.

(B) Mean peak amplitudes of responses (control: n = 364 synapses from 7 coverslips; 500 pM $A\beta_o$: n = 245 synapses from 7 coverslips; 10 nM $A\beta_o$: n = 166 synapses from 7 coverslips; 200 nM $A\beta_o$: n = 323 synapses from 7 coverslips). ANOVA with post-hoc *t*-test and Dunnett's correction.

(C) Representative images showing hippocampal neuronal boutons expressing vGAT-pHluorin under control of the GAD67 GABAergic neuron-specific promoter ($GAD67^{pro}$-vGAT-pH) and incubated for two hours with either 200 nM $A\beta_o$ or vehicle control. Middle panels show the increase in fluorescence after stimulation at 10 Hz for 10 seconds. Right hand side panels show maximal signal following unquenching of vGAT-pH with NH_4Cl, used for normalization as a control for vGAT-pH expression level. Scale bar = 5 μm.

(D) Average $GAD67^{pro}$-vGAT-pH fluorescence traces during 10 Hz/10 s stimulation.

(E) Mean peak amplitudes of 10 Hz/10 s responses (control: n = 50 synapses from 5 coverslips; 200 nM $A\beta_o$: n = 90 synapses from 6 coverslips).

(F) Fluorescence traces showing mean 10 trial average SypH 2x responses to a single AP stimulus (arrow) before and after standard treatment with the Ca^{2+} chelator EGTA-AM (200 μm for 90 s).

(G) Mean post-treatment response expressed as % of mean pre-treatment response (control: n = 155 boutons from 8 coverslips; $A\beta_o$: n = 137 boutons from 7 coverslips).

(H) SNX-482, a blocker of $Ca_V2.3$, rescues the effect of $A\beta_o$ on neurotransmitter release in SypHy-expressing neuronal cultures while the mGluR5 blocker SIB-1757 does not. Responses to a 100 AP/10 Hz stimulus are normalized to maximal NH_4Cl signal and mean peak amplitudes are shown. Average fluorescence traces are above bars (vehicle-treated control: n = 126 boutons from 5 coverslips; $A\beta_o$: n = 69 boutons from 5 coverslips; SNX-482: n = 54 boutons from 5 coverslips; $A\beta_o$ + SNX-482: n = 50 boutons from 5 coverslips; SIB-1757: n = 90 boutons from 5 coverslips; $A\beta_o$ + SIB-1757: n = 95 boutons from 5 coverslips). ANOVA with post-hoc *t*-test and Sidak correction.

(I) Synaptosomes were prepared from individual hippocampal neuronal cultures before each synaptosome preparation was divided and subjected to treatments as indicated. PKC activity was then assessed in synaptosomal lysates using a specific ELISA-based assay (n = 10 cultures for all conditions). Paired *t*-test.

Shading or error bars represent ± s.e.m. $^{***}p < 0.0001$, ns = non-significant.

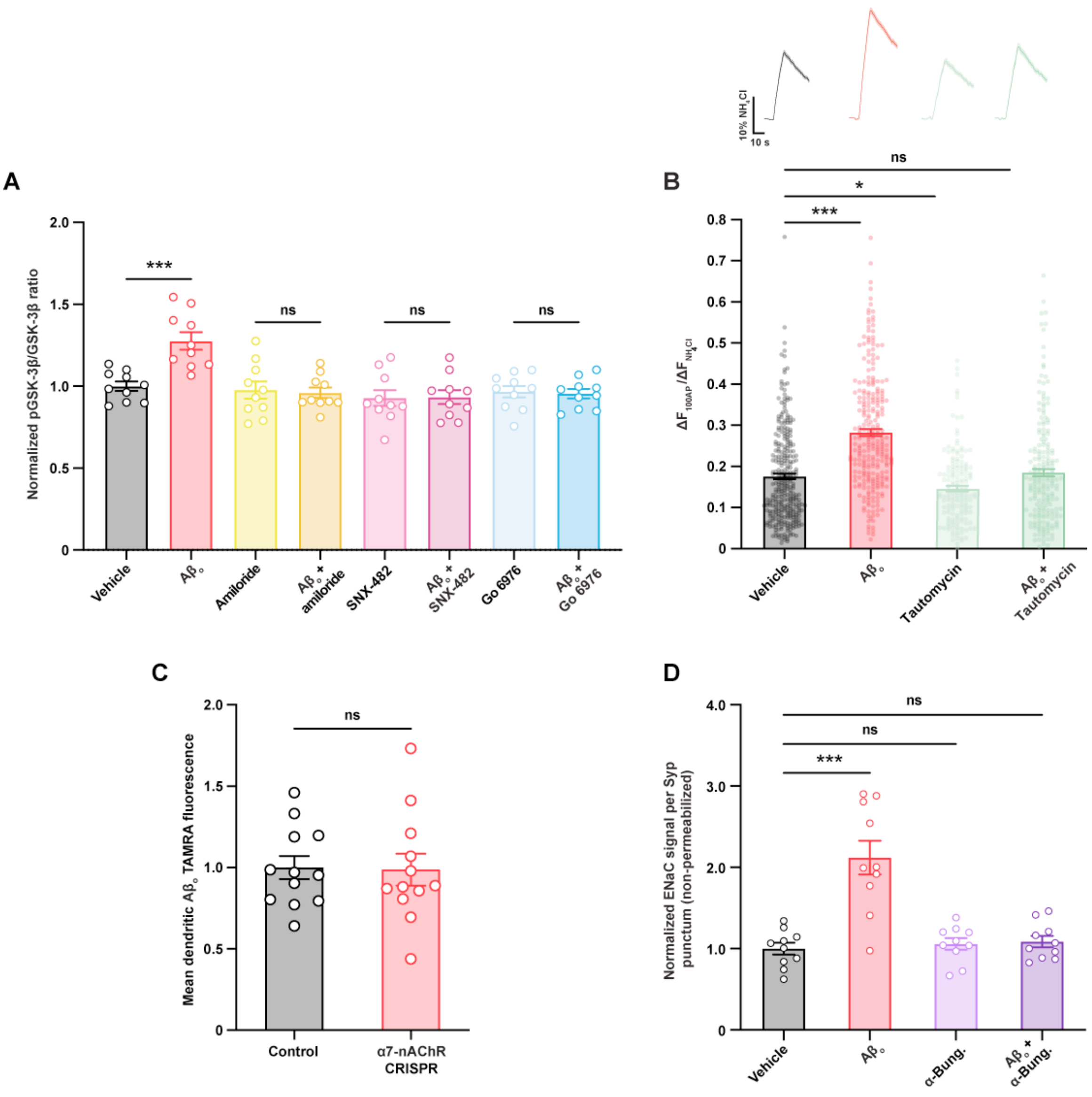

Figure S3. Related to Figures 2 & 3.

(A) Immunofluorescence for total GSK-3β, GSK-3β that has been inactivated by phosphorylation of the serine 9 residue and synatophysin was carried out on dissociated hippocampal neurons treated as indicated. To examine presynaptic GSK-3β specifically, only labelling overlapping with synaptophysin was assessed. Graph shows the fraction of GSK-3β that has undergone inactivating phosphorylation at S9 represented as the S9 phosphorylated : total GSK-3β signal intensity ratio normalized to control average (n = 10 fields from 5 cultures per condition).

(B) The PP1/PP2a antagonist tautomycin rescues $A\beta_o$-induced enhancement of synaptic vesicle exocytosis. Mean peak amplitudes of SypHy responses to a 100 AP/10 Hz

stimulus train following the indicated treatments (vehicle-treated control: n = 324 synapses from 7 coverslips; $A\beta_o$: n = 273 synapses from 7 coverslips; vehicle + tautomycin: n = 163 boutons from 5 coverslips; $A\beta_o$ + tautomycin: n = 206 boutons from 5 coverslips).

(C) Normalized mean dendritic fluorescence intensity in control and α7-nAChR knockout neurons incubated with $A\beta_o$ TAMRA (n = 12 neurons for both conditions).

(D) Immunofluorescence for ENaC and synaptophysin was carried out on non-permeabilized dissociated hippocampal neurons treated as indicated. Total amount of presynaptic membrane-inserted ENaC was assessed as ENaC labelling intensity per synaptophysin-positive punctum (n = 10 cells per condition).

Error bars represent ± s.e.m. $^{*}p < 0.05$, $^{***}p < 0.0001$, ns = non-significant.

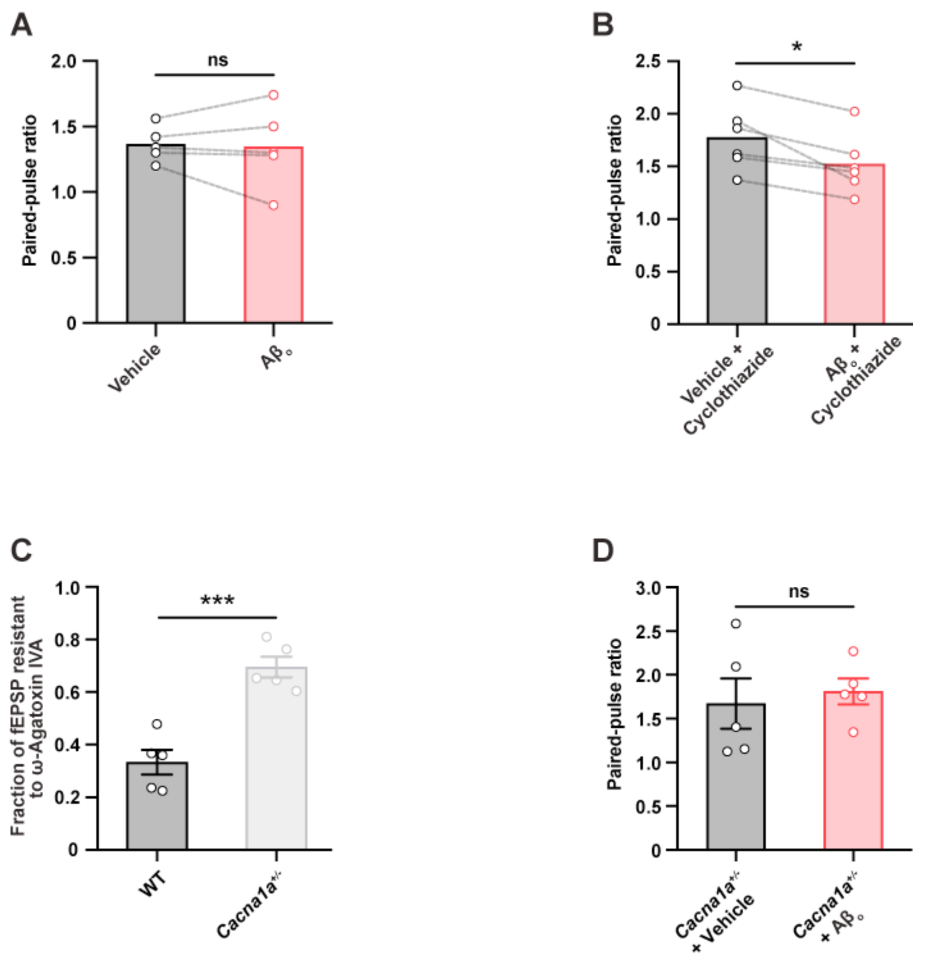

Figure S4. Related to Figures 4 & 5.

(A) Paired-pulse ratio (PPR) with 50 ms interpulse interval measured at CA3-CA1 synapses in acute hippocampal slices treated as indicated. $A\beta_o$ were applied at 10 nM (both groups n = 5 paired slices from 5 mice). Paired *t*-test.

(B) PPR with 50 ms interpulse interval measured in acute hippocampal slices treated as indicated (10 nM $A\beta_o$). Cyclothiazide (100 μM) was added to prevent AMPA receptor desensitization, which can confound PPR measurements in the presence of $A\beta_o$ (both groups n = 6 paired slices from 6 mice). Paired *t*-test.

(C) Stable baseline synaptic transmission was recorded from CA3-CA1 synapses of wild-type or *Cacna1a*$^{+/-}$ hippocampal slices before ω-agatoxin IVA was added to the perfusing ACSF. Agatoxin resistance was calculated by comparing the average slopes of fEPSPs during 5 minute windows immediately before and 10 minutes after toxin addition (both genotypes n = 5 slices from 5 mice).

(D) *Cacna1a*$^{+/-}$ hippocampal slices are resistant to the effects of 10 nM $A\beta_o$ on PPR with 50 ms interpulse interval recorded in the presence of cyclothiazide (both groups n = 5 slices from 5 mice).

Error bars represent ± s.e.m. $^{*}p < 0.05$, $^{***}p < 0.01$, ns = non-significant.

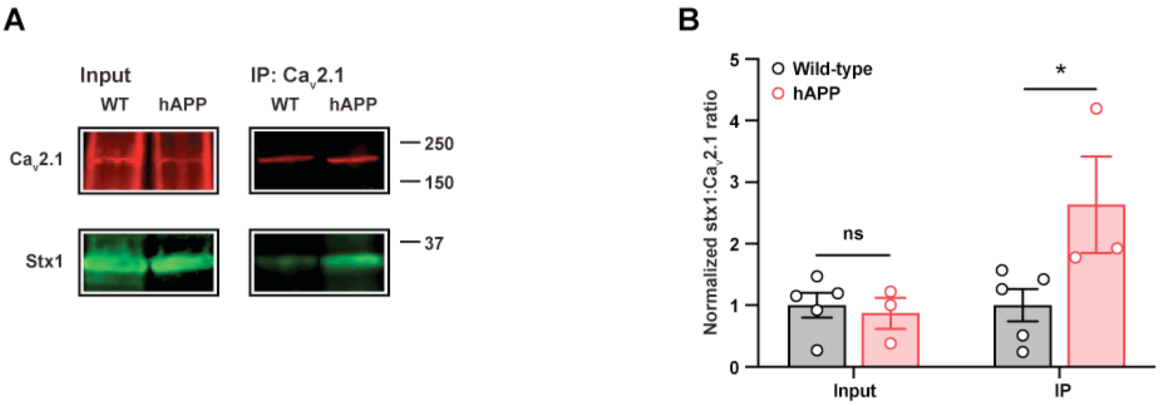

Figure S5. Related to Figure 5.

(A) The $Ca_V2.1$ α_{1A} subunit was immunoprecipitated from purified synaptosomal membrane fractions prepared from frontal cortex of 20 month old hAPP mice and littermate controls. Following SDS-PAGE separation, samples were probed with $Ca_V2.1$ and syntaxin 1 (Stx1) antibodies as indicated. Panels show representative bands from both input samples and immunoprecipitates.

(B) Raw immunoblot Stx1 : $Ca_V2.1$ signal ratios from each individual were normalized to the control group mean. Enhanced Stx1 : $Ca_V2.1$ ratio in immunoprecipitated samples indicates stronger interaction between $Ca_V2.1$ and syntaxin 1 in brains of hAPP mice (wild type: $n = 5$; hAPP: $n = 3$). Repeated measures ANOVA with post hoc *t*-test and Sidak correction.

Error bars represent ± s.e.m. $^{*}p < 0.05$.

Internal reference	Age	Sex	Pathological diagnosis
**Control**			
A407/13	80	F	Control - consistent with aging, Braak stage 2
A002/13	90	M	Control - mild age-related changes. Modified Braak stage 1 with mild focal amyloid angiopathy
A114/12	82	M	Control - aging process, consistent with Braak stage 2
A046/12	92	F	Control - modified Braak stage 2
A359/08	80	F	Control - minimal aging changes consistent with BrainNet Europe (BNE) stage 1; childhood poliomyelitis
**Alzheimer's**			
A374/13	78	F	Alzheimer's disease - BNE stage 6
A308/13	74	F	Alzheimer's disease - BNE stage 6
A348/12	75	M	Alzheimer's disease - BNE stage 6 with mild amyloid angiopathy
A244/12	75	M	Alzheimer's Disease - modified Braak stage 6 with focal amyloid angiopathy and neocortical TDP-43 pathology
A187/12	94	F	Alzheimer's disease - BNE stage 6

Table S1. Related to Figure 5.
Patient data for human brain samples used in this study.

Supplemental References

S1. Velasco, P.T., Heffern, M.C., Sebollela, A., Popova, I.A., Lacor, P.N., Lee, K.B., Sun, X., Tiano, B.N., Viola, K.L., Eckermann, A.L., et al. (2012). Synapse-binding subpopulations of Abeta oligomers sensitive to peptide assembly blockers and scFv antibodies. ACS Chem Neurosci *3*, 972-981. 10.1021/cn300122k.
